# A Survey on the Taxonomy of Cluster-Based Routing Protocols for Homogeneous Wireless Sensor Networks

**DOI:** 10.3390/s120607350

**Published:** 2012-05-30

**Authors:** Soroush Naeimi, Hamidreza Ghafghazi, Chee-Onn Chow, Hiroshi Ishii

**Affiliations:** 1 Department of Electrical Engineering, University of Malaya, Kuala Lumpur, 50603, Malaysia; E-Mail: cochow@um.edu.my; 2 School of Electrical Engineering and Computer Science, University of Ottawa, Ottawa, ON K1N 6N5, Canada; E-Mail: hghaf099@uottawa.ca; 3 School of Information and Telecommunication Engineering, Tokai University, Tokyo 259-1292, Japan; E-Mail: ishii@ishiilab.net

**Keywords:** wireless sensor networks, homogeneous networks, clustering protocol, cluster head, cluster formation, data aggregation, failure management, resource-aware design, survey

## Abstract

The past few years have witnessed increased interest among researchers in cluster-based protocols for homogeneous networks because of their better scalability and higher energy efficiency than other routing protocols. Given the limited capabilities of sensor nodes in terms of energy resources, processing and communication range, the cluster-based protocols should be compatible with these constraints in either the setup state or steady data transmission state. With focus on these constraints, we classify routing protocols according to their objectives and methods towards addressing the shortcomings of clustering process on each stage of cluster head selection, cluster formation, data aggregation and data communication. We summarize the techniques and methods used in these categories, while the weakness and strength of each protocol is pointed out in details. Furthermore, taxonomy of the protocols in each phase is given to provide a deeper understanding of current clustering approaches. Ultimately based on the existing research, a summary of the issues and solutions of the attributes and characteristics of clustering approaches and some open research areas in cluster-based routing protocols that can be further pursued are provided.

## 1. Introduction

Recent advances in Micro-Electro-Mechanical Systems (MEMS) in tandem with significant developments in digital signal processing (DSP) have led to the great development of micro-sensors. While in the past the wired sensors were implemented in limited applications in industries, wireless implementation makes the wide deployment of sensor nodes more feasible than before. In the past decade, there has been much research regarding the great potential capabilities of wireless sensor networks (WSNs) in applications such as environmental monitoring, habitat study, military surveillance in the battlefield and home automation. With sharp decreases in cost and tangible improvements in storage and processing capabilities of sensor nodes, the integrated presence of sensor nodes in human everyday-life, as the connector of the physical environment with virtual digital world, will be dominant in near future. Vast deployment of nodes on large-scale dimensions entails deep investigation on routing protocols to ensure reliable and real-time data transmission, while considering the power constraints inherent in WSNs. Normally, a sensor node is powered by a battery, and is unattended once deployed, therefore the proposed routing protocols for WSNs should not only address the challenges regarding the Quality of Service (QoS) of the application such as real-time operation, fault tolerance, scalability and data reliability, but the limited capabilities of WSNs in energy storage, processing, memory and communication and topology changes due to nodes' mobility and demises should be addressed too.

Given the unique characteristic of WSNs, cluster-based protocols show significant advantages over flat strategies. Followings are several advantages of clustering schemes that introduce them as the most compatible protocols with WSNs attributes:

- Minimizing the total transmission power.
- Balancing the energy-exhausting load among all nodes.
- Reducing the bandwidth demand and efficient use of limited channel bandwidth.
- Lessening routing and topology maintenance overhead.
- Eliminating the redundant and highly correlated data in aggregation process.
- Reducing data collision and interference in data transmission process by use of multi-power levels in cluster-scale and network-scale communications.
- Localizing the route setup within the cluster boundaries and thus generating small-size routing tables.
- Increasing the manageability and scalability of the network.

**Figure 1.**
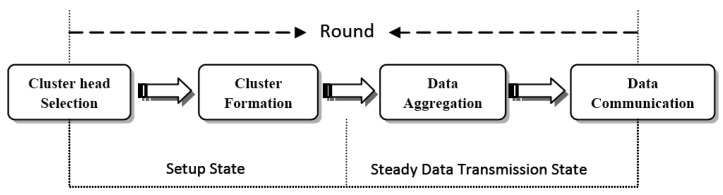
The composition of one round of the clustering process.

Cluster-based routing protocols consist of four stages: cluster head selection, cluster formation, data aggregation and data communication. As it is seen in Figure 1, the setup state starts by the cluster head selection stage and proceeds by constructing clusters. The setup state is followed by the steady data transmission state, which is subdivided into data aggregation and data transmission phases. The setup and steady data transmission states form one round of running a cluster-based protocol, which iterates throughout the time of running the protocol or the network lifetime. Based on the role, sensor nodes in clustering algorithms may be grouped into four categories:

- *Cluster head (CH)*: Coordination of a group of nodes located within the boundaries of the cluster, aggregating the sensed data by the cluster members and transmission of the aggregated data to the next hop are the main duties of a CH.
- *Base station (BS)*: Given the high processing capabilities and unlimited source of energy, BS may be the coordinator of the network and/or the sink node where all the aggregated data are processed according to the type of the application and demands of the end user.
- *Relay node (RN)*: Groups of nodes in multi-hop data transmission schemes responsible for relaying sensed or aggregated data by other nodes towards the destination
- *General node (GN)*: Majority of nodes in the network, which only provide the sensed data based on the type application.

Several existing surveys on the cluster-based routing protocols for WSNs can be found in the literature [1–9]. The articles [1–3] survey the strengths and weaknesses of limited numbers of existing clustering protocols separately, without providing any classification of them. Abbasi and Younis in [4] give a taxonomy of the different attributes of clustering algorithms, which are classified and evaluated according to their convergence rate into two groups of variable and constant convergence time. Dechene *et al.* in [5] group clustering algorithms into four schemes: heuristic, weighted, hierarchical and grid. The paper also reviews and compares limited numbers of clustering algorithms for each scheme in detail. The authors in [8] provide an insight into routing protocols designed specifically for large-scale WSNs. By focusing on energy efficiency as a problem of great significance in large-scale networks, the article categorizes the algorithms based on the motivation of the methods for improving energy efficiency as control overhead reduction, energy consumption mitigation and energy balance. However, all the aforementioned surveys give a summary of the limited famous routing protocols and compare their attributes without focusing on the limitations that exist in separate phases of a clustering algorithm in homogeneous networks. To the best of our knowledge, the work presented in this paper is the first and the most comprehensive survey, which covers and analyzes a large-number of recent available literatures on cluster-based routing protocols for homogeneous networks according to their contributions in each individual phase of CH selection, cluster formation, data aggregation and data communication. Besides, the paper classifies the schemes based on their main objectives and contribution towards addressing the shortcomings of each phase of clustering process. Furthermore, we believe this paper serves as a useful starting point for the researchers who are interested in conducting research in clustering algorithms. A list of symbols in accordance with the occurrence of the symbols in equations is provided in Table 1. To eliminate the ambiguity, the symbols of common parameters in different equations are unified and presented in the table based on their occurrence in equations.

This paper is organized in the following way: the taxonomy and the state of the research of the setup phase in cluster head selection and cluster formation stages are presented in Section 2 and Section 3, respectively. Section 4 investigates data aggregation process in details in homogeneous cluster-based schemes. In Section 5, different strategies in the data transmission phase are surveyed and analyzed. Finally, Section 6 summarizes the issues and solutions of the attributes and characteristics of clustering approaches and some related open research areas for the design of cluster-based routing protocols are presented too.

## 2. Cluster Head Selection

**Figure 2.**
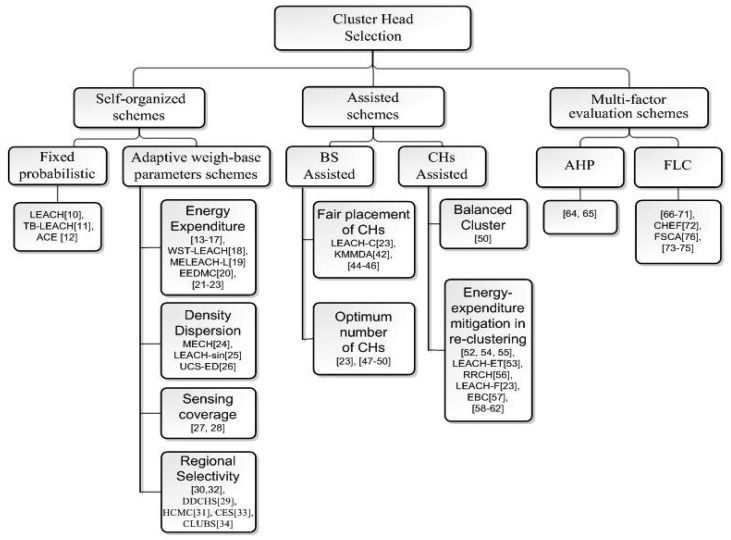
The taxonomy of CH selection.

The first step in cluster-based protocols is the selection of CHs. CH as the local coordinator or sink of the cluster handles numerous tasks of coordination of the work between node members, collection of information within the cluster, data fusion processing and transmission of the aggregated data towards the global sink. In addition, even division of the nodes into clusters is dependent on the number and location of the CHs. Therefore, CH selection plays a significant role in the subsequent procedures of a clustering algorithm and thus performance, lifetime and energy-efficiency of the network. Due to this importance, many researchers have focused on optimizing the CH selection process. Based on the different strategies implemented in CH selection processes, we classify them as self-organized schemes, assisted schemes and multi-factor evaluation schemes. Figure 2 shows the taxonomy of CH selection.

### 2.1. Self-Organized Schemes

In these schemes, the CH selection decision is distributed within the network and each node. Either stochastically or involving some probabilistic parameters, nodes compete to be selected as CH. Based on an absolute stochastic selection or involving some resource parameters, self-organized CH selections can be grouped into two categories: fixed probabilistic schemes and adaptive weight-based parameters schemes.

#### 2.1.1. Fixed Probabilistic Schemes

In absolute probabilistic schemes, nodes make autonomous decisions without any centralized control. CHs are selected for initial and subsequent rounds only based on the evaluation of an expression that includes some fixed parameters like number of CHs, current round number, time interval or Node ID. The first and the most popular self-organizing clustering protocol for WSNs, called Low-Energy Adaptive Clustering Hierarchy (LEACH) [10] takes advantages of randomization to evenly distribute the energy expenditure among the nodes within the network. In LEACH, each node creates a random number between [0, 1] and compares it with a threshold value T(n), calculated by the Equation (1), to determine whether it is chosen as CH role in current round or not. The node becomes a CH if this number is lower than T(n). If a node is selected as CH, it broadcasts the CH advertisement messages within the network and other nodes join the cluster based on the received signal strength.

(1) $$T\left(n\right)=\frac{P_{CH}}{1-P_{CH}*\left(r_{c}\;\text{mod}\;1/p_{CH}\right)}$$

where *P_CH_* is the desired percentage of CHs, *r_c_* is the current round, and *n* is the nodes that have not been CH in the last 1/p rounds [10].

Keeping the stochastic notion of CH selection strategy in LEACH, Time-Based CH selection (TB-LEACH) [11] proposes that the competition for CH position no longer be dependent on a random number but a random time interval, in which nodes having the shortest time interval win the competition for the CH role. According to the scheme, every node sets a random-interval timer at the outset of each round, when the timer expires, the nodes, which have not received the predefined number of CH advertisement messages, broadcast a CH advertisement throughout the network. To have constant number of CHs, a counter is also set that stops the CH competition when the number of selected CHs reaches the desired value. However, the counter works precisely only if all nodes are in the broadcast range of CH advertisement of all the CHs, which may not be a true presupposition in large scenarios with far distances between nodes.

Unique identifier (ID) of the nodes and desired number of CHs are the parameters considered by [12] to present two deterministic schemes of Algorithm of Cluster-head Election by Counting (ACE-C) and Location (ACE-L). In ACE-C, the CH selection for a network consisting of *N* sensor nodes is based on round-robin fashion. Where number of desired CHs is supposed as *C*, the sensor nodes with ID's from 0 to *C − 1* in first round, and nodes with ID's from *C* to *2C − 1* in next round are selected CHs. The same procedure continues for following rounds until all the nodes become CH once and whole process starts again from the node with ID = 0, ultimately. To make the CH decision approach distributed, each node considers the total number of selected CHs and its ID in each round to announce its selection as CH in its turn. ACE-L, which is especially proposed for mobile sensor nodes, uses location information presented as some fixed reference points to select the predefined number of CHs. The algorithm selects constant number of reference points equal to the desired number of CHs, and each node selects the closest reference point as its main reference point (MRP). The nodes with the same MRP contend a channel for the CH role based on the metric of the *delay time*, which is directly proportional to distance of the nodes to reference point. In other words, the node closer to the reference point broadcasts a beacon of being CH earlier and other nodes by receiving the beacon stop the competition.

#### 2.1.2. Adaptive Weight-Based Parameters Schemes

In clustering algorithms, CHs as the local coordinator of the clusters play the key role in network performance. Therefore, from a local perspective, the lifespan and performance of a part of the network, which is coordinated by a CH, is quite related to the accessibility and performance of its CH. On a network scale, the relative position and even distribution of CHs according to the node density in the network field are important features that greatly affect the network throughputs. Although the selection of the CH in adaptive weight–based schemes [13–22] is still distributed among the sensor nodes, the procedure is not based on sheer probabilistic solutions anymore, and rather node resources and other determinant factors are weighed up in selection of the optimum CHs. In this respect, we classify these schemes in accordance with the factors that are considered to improve the stochastic CH selection schemes as energy expenditure, density dispersion, sensing coverage and regional selectivity.

##### 2.1.2.1. Energy Expenditure

The node selected as CH consumes more energy than other nodes within the network and demise of a CH leads up to losing all the data of an area monitored by its cluster member nodes. Therefore, selection of the nodes having the highest remaining energy as CH is quite desirable [13–17]. Considering a ratio of the current level to the initial energy level of the node as a coefficient of the probability equation of the threshold value T(n) is proposed in [14,23]. The nodes with higher level of remaining energy have more chance to be selected as CH. However, the average energy of the nodes decreases after certain number of rounds. Therefore, multiplying T(n) with a small fraction lessens the chance of nodes to be selected as CH and may lead to the rounds with few or even no CHs, although there are still nodes available having enough battery power to communicate with the BS and are able to play the CH role. The possible solution for the issue is proposed in [14] by normalizing the energy coefficient, presented in Equation (2):

(2) $${\text{T}}^{\prime }\left(\text{n}\right)=\{\begin{array}{c} \frac{{\text{P}}_{\text{CH}}}{1-{\text{P}}_{\text{CH}}*\left({\text{r}}_{\text{c}}\;\text{mod}\;\frac{1}{{\text{P}}_{\text{CH}}}\right)}\left[\frac{{\text{E}}_{\text{c}}}{{\text{E}}_{\text{i}}}+\left({\text{r}}_{\text{c}}\;\text{div}\frac{1}{{\text{P}}_{\text{CH}}}\right)\left(1-\frac{{\text{E}}_{\text{c}}}{{\text{E}}_{\text{m}}}\right)\right],\text{if n}\in \text{G}\\ 0,\text{otherwise} \end{array}$$

where *E_c_* is the current energy and *E_i_* is the initial energy of node *n*. When *r* reaches the value *1/P* the threshold *T(n)* is reset to the value it had before the inclusion of the remaining energy into the threshold-equation. Hence, by decreasing the average energy level of the nodes, the chance being a CH is not eliminated

In [13], the paper proposes a modified version of threshold equation for CH selection based on the square of the ratio of the current to the initial level of energy of the nodes. According to the Equation (3), when the node *n* has more energy, the influence of the energy ratio is relatively large, and by depleting the battery power of the node, the effects of the energy ratio factor are diminished too. Therefore, the proposed scheme also mitigates the problem of decrease in the probability chance of individuals by depletion of their battery power:

(3) $$T^{\prime }\left(n\right)=\frac{{\text{p}}_{\text{CH}}}{1-{\text{p}}_{\text{CH}}*\left({\text{r}}_{\text{c}}\mathrm{mod}1/{\text{p}}_{\text{CH}}\right)}*\sqrt{\frac{E_{c}}{E_{i}}}$$

The scheme presented in [16], in addition to the current energy level of the nodes, considers three other factors in the last round including the initial energy level, the total dissipated energy level of a node and the initial average remaining energy level of all the nodes within the cluster. In other words, the main idea of the algorithm is to choose nodes with higher energy level and lower energy dissipation as CHs. Based on Equation (4), nodes having the energy level lower than the average energy level of the cluster and even nodes with higher energy level but consuming relatively more energy in the last round have lower chances to be selected as CH. Adding the dissipated energy of the nodes in last round can further regulate the variation speed of T'(n):

(4) $$T^{\prime }\left(n\right)=\frac{p_{CH}}{1-p_{CH}*\left(\left(r+1\right)\text{mod}\frac{1}{p_{CH}}\right)}\left[\frac{E_{r}-E_{r\_\text{dissipate}}}{E_{r\_\text{average}}-E_{r\_\text{dissipate}}}\right]$$

where E_r_ is the node residual energy in the beginning of the last round *r, E_r_averge_* is the average initial energy of all nodes within the cluster in the last round *r* and *E_r_dissipate_* is the node total dissipated energy for data transmission during last round *r*.

Cluster-Chain Routing Protocol (CCRP), proposed in [15], uses an additional factor of the number of the neighbours to increase the chance of the nodes having more neighbours as being CH. Thus, the algorithm limits the intra-cluster communication cost of cluster members with the CH:

(5) T'(n)={PCH1−PCH∗(rmod1PCH)[EcEi+(1−EcEi)Nb1/PCH−1],if n∈G0,otherwise

where *N_b_* is the number of the neighbours and other variables are the same as what they are in Equation (2).

In [18], Weighted Spanning Tree for LEACH (WST-LEACH) is proposed that is based on a weighted consideration of the remaining energy, distribution density and the distance of nodes from the BS. In a network with *N* nodes, the algorithm considers variant weights for three following ratios as a coefficient to be applied to T(n): ratio of the residual energy E_residual_ to initial energy *E_0_*, ratio of the number of neighbours N_b_ in predefined radius R to the average number of nodes in accordance with the probability *P_r_* and inverse correlation of the nodes' distances from the BS (d_BS_). The modified threshold value in Equation (6) improves the stochastic method of CH selection in LEACH to a multi-criteria decision-making process. However, the appropriate selection of the ratio weights is essential in generating an efficient threshold value, which does not degrade the network performance either by decreasing the average chance of the nodes to be selected as CH or by proliferation of the network with redundant number of CHs:

(6) $$T^{\prime }\left(n\right)=\frac{{\text{P}}_{\text{CH}}}{1-{\text{p}}_{\text{CH}}*\left(\text{r mod}\;1/{\text{P}}_{\text{CH}}\right)}\times \left\{{\text{W}}_{1}*\frac{E\_\text{residual}}{E_{0}}+{\text{W}}_{2}*\frac{N_{b}}{p_{r}*N}+{\text{W}}_{3}*\frac{1}{d_{BS}}\right\}$$

Other than selection of CHs through modification of LEACH probabilistic threshold scheme, there are some algorithms [19,20] in which CHs are chosen according to a time contest. In these schemes, the CH selection procedure is through participation in a contest for the broadcast time of the CH advertisement by setting up a random timer according to their resource information. The nodes of which their timers expires earlier, broadcast the CH advertisement message, while other nodes that receive the advertisement messages and their timers are not expired, drop the competition for CH role and join the CH with the minimum communication cost. The timer in More Energy-efficient LEACH for Large-scale sensor networks (MELEACH-L) algorithm [19] is a function of residual energy of nodes and duration of the CH selection phase. As it can be seen in Equation (7), the timers of the sensor nodes having higher energy level expire earlier at higher probability, and hence the nodes with lower battery power level close to high-energized nodes have little chance to be as CH:

(7) $$T_{i}=\left[\alpha_{c}\frac{E_{\text{start}}-E_{\text{residual.i}}}{E_{\text{start}}}+\left(1-\alpha_{c}\right)\text{random}\left(0,1\right)\right].\delta$$

Where α_c_ is a constant, which determines the weight of the energy resource and random number in lateness of the timer, δ is the time duration of the CH selection phase.

In the Energy-Efficient Distributed Multi-level Clustering (EEDMC) algorithm [20], at the beginning of each round, each node broadcasts a message to the neighbouring nodes consisting of their node IDs and residual energy. In this way, each node saves a table of its neighbouring nodes and their remaining energy, and calculates the average residual energy of its neighbours as a parameter to generate the timer value by Equation (8):

(8) $${\text{t}}_{\text{i}}=\text{p}\times \text{T}\times \left(1-\frac{\sum_{j=1}^{m}E_{rj}}{N_{b}}\right)$$

where *p* is a random number between [0.95, 1] to avoid advertisement message collision of the nodes with the same remaining energy, *T* is the predefined maximum time of CH competing duration, *N_b_* is the number of neighbouring nodes and *E_rj_* is the residual energy of the neighbouring node *j*. Equation (8) gives negative results for the average energy level over one unit, hence, to avoid this, the remaining energy level of the nodes should be normalized by their initial energy level.

##### 2.1.2.2. Density Dispersion

The dispersion of CHs should conform to the nodes' density in distribution; in other words, selection of the nodes from denser areas leads to conserving more energy. Ruay *et al.* in [24] present a mechanism to select the Maximum Energy Cluster-head (MECH) based on the number of neighbours of the node. Every node broadcasts a Hello message to its one-hop neighbours and in this way, each node counts the number of its neighbours. When the number of the received messages reaches a specified value, the node announces its selection as CH by broadcasting an advertisement to its one-hop neighbours. The nodes that receive the advertisement set a back-off timer and never broadcast the same message. By expiring the back-off time, every node selects the nearest CH based on the signal strength. The method ensures the existence of one CH in a region consisting of the neighbouring nodes with a predefined communication range. However, the process of detecting the neighbouring nodes imposes extra control messages to the network and delays the network convergence time in proportion to back-off time.

LEACH-sin [25] focuses on asymmetrical distribution of CHs in network area and changes the probability of being CH in sinusoidal circle around the sink. In this respect, an adjustment function is introduced in Equations (9) and (10) to change the distribution of the threshold value of the nodes within the network. Round number *r_c_*, distance of the nodes from the BS (d_BS_) and the optimum cluster radius *R* are the variables considered in the adjustment function. Multiplication of T(n) by f(r_c_, d_BS_) gives a new distributed threshold value, which changes symmetrically over the rounds of running the protocol:

(9) $$\text{f}\left({\text{R}}_{\text{n}}{,\text{d}}_{\text{bs}}\right)=\left|\sin(\frac{\pi }{2\text{R}}({\text{d r}}_{\text{c}}\mathrm{mod}\left(\frac{1}{\text{p}}\right).2\text{pR}\right|$$

(10) $$T^{\prime }\left(n\right)=\frac{\text{p}}{1-\text{p}*\left({\text{R}}_{\text{n}}\;\text{mod}\;1/\text{p}\right)}*\text{f}\left({\text{r}}_{\text{c}}{,\text{d}}_{\text{BS}}\right)$$

Since the absolute value of the sinusoidal adjustment-function is always equal or less than one, applying the function to threshold value decreases the number of the CHs over running rounds. Hence, the paper calculates the coefficient of π/2 to apply to the desired number of CHs to compensate the diminution of the threshold caused by the adjustment function.

In [26], the authors propose an Uneven Clustering Scheme based on the Energy and Distribution density of CHs (UCS-ED), which is an application oriented algorithm that especially addresses asymmetric data throughput in underground area in coal mine. Since in a laneway area of a coalmine, the direct transmission of the data from far CHs to the BS is not possible, CHs in addition to data aggregation and transmission to the BS are also a backbone to relay aggregated data of other CHs. Therefore, the CHs nearer to the BS deplete at a faster rate. Hence, to mitigate this issue, the paper proposes a CH selection algorithm that the regions closer to the BS generate more CHs than the further regions. In this respect, at the initial stage, the BS broadcast a “Hello” message to all nodes and in this way, each node can evaluate the received signal strength and estimates its approximate distance from the BS. Then, the nodes having the higher remaining energy and closer to the BS have higher chances to be selected as CH and so, the density of the CHs follows the effective density of backbone.

##### 2.1.2.3. Sensing Coverage

As the main objective of implementing sensor nodes is to sense a physical phenomenon, avoiding coverage holes within the network should be a top priority. In [27], the paper proposes coverage area in addition to residual energy as combined metrics for the selection of CHs. The algorithm divides the sensor nodes into two groups of normal and critical nodes. The nodes with overlap coverage percentage over a threshold value (70% proposed by the paper) by other sensor nodes are considered as normal nodes and have normal sleeping intervals, but the nodes with less overlap percentage are marked as critical nodes and have longer sleeping intervals. To determine the overlap coverage percentage, each node, at the initial phase, broadcasts a message with the transmission power equal to its sensing range (usually considers as half the transmission range) and the nodes, which receive the message, response to the message during a certain time period. In this way, each node calculates its overlap coverage percentage. In CH selection phase, each node sets a timer based on remaining power and coverage overlap and thus the nodes having the greater residual energy and more overlap coverage have more chances to be selected as CH. The critical nodes do not participate in CH competition to retain the sensing coverage level of the network, even if they have more battery power than normal nodes.

Another algorithm that considers the sensing coverage is presented in [28]. The paper calculates the estimated normalized effective sensing area of the nodes through finding the overlapped sensing nodes. Each node broadcasts a beacon message to its neighbouring nodes to discover them. The transmission power of the beacon message is set to reach the nodes within two sensing range times. Therefore, each node can find its overlapped sensing area and estimates its effective sensing range. It is concluded that demise of the nodes with larger value of effective sensing range causes greater coverage hole and further degrades the network performance. Hence, the algorithm multiplies the original probability in LEACH by the estimated normalized overlapping area parameter. Thus, the nodes with large value of effective sensing range have less probability to be selected as CH. In other words, the CH role is mainly carried by nodes with small normalized overlapping area values.

##### 2.1.2.4. Regional Selectivity

In some other algorithms [29–34], CH selection is not the initial stage of the clustering algorithm. On the contrary, first each sensor node finds its neighbouring nodes in a predefined radius or number of hops, or performs a rudimentary regional cluster formation stage based on the position of the nodes within the network, and then the most qualified node in each section is selected as CH based on a distributed algorithm. This method improves the evenness of CH dispersion within the network and eliminates re-clustering procedures in network dimensions by rotating the CH role among the nodes within a certain region. However, in distributed algorithms, determining the neighbouring nodes and gathering their resource information delays completion of the setup phase and imposes extra network implementation costs by utilizing localization algorithms [35–40] or equipping the nodes with localization equipment such as Global Positioning Systems (GPS). Moreover, the decision based on the local information does not necessarily provide the most optimized selection from a network scale perspective.

Density and Distance based Cluster Head Selection (DDCHS) that divides cluster area into two perpendicular diameters to get four quadrants is proposed in [29]. By grouping the nodes, the node density in each quadrant is compared and the candidate quadrants are selected. Then based on the distance of the candidate quadrants from the cluster centre, the nearest one is selected as the CH of the quadrant. In [30] the authors also divide nodes into several circular tracks around the BS and in each track, nodes having more energy level and less distance from higher-level CHs are selected to play the CH role.

By using the Monte-Carlo localization Box-Redrawing (MCBR) built upon MCB [40], the paper [31] presents the Weighted Clustering algorithm based on Monte-Carlo localization clustering scheme. The algorithm classifies nodes into hexagons based on their distances from their neighbouring nodes. In the next step, the CH is selected in each hexagon according to the weights of the nodes. A four weighting-factors formula is presented, including distance of the node to the centre of the corresponding hexagon, total time in which node has acted as CH, average distance between any two nodes belonging to the same hexagon and the percentage of energy consumed by the node. Nodes with the least weight value are selected as CHs. The localization algorithm proposed by the paper uses some mobile anchors to help in localizing nodes, which is not feasible in all scenarios such as battlefields or harsh environment monitoring.

**Figure 3.**
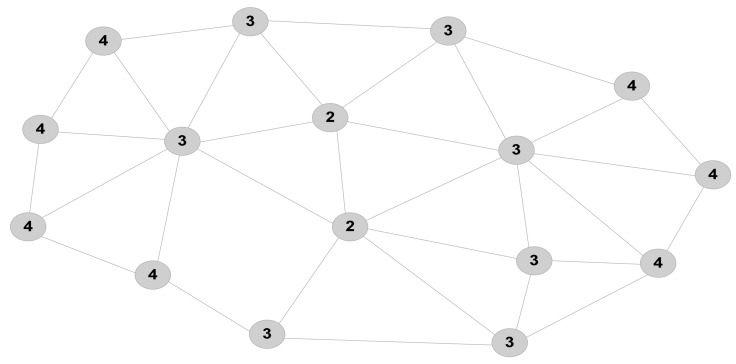
Nodes with Max-min hops to any other sensor nodes.

The work presented in [32] proposes an algorithm to select suitable CHs which cover more regions with the smallest average total communication distances. The paper uses the maximum number of minimum hops (Max-min hops) to find nodes located at the centre of the network. In this respect, every node generates a list of its neighbouring nodes, including their minimum hop number distances. When each sensor node has the min-hop number to all other nodes, it finds the maximum hop (Max-hop) number among them and exchanges the value with other nodes within the network. It is seen in Figure 3 that nodes with the minimum Max-hop numbers are located in central area of the network, and thus, are more eligible to be selected as CH. However, the downside of the algorithm is that it may lead to having CHs not adequately apart from each other. To address this issue, the paper includes the distances of CHs from each other as another determinant factor in selection of CHs. Moreover, the cost of broadcasting the heuristic messages and the convergence time of the algorithm in large scenarios with numerous numbers of nodes is not well suited with the constraint resources and real-time applicability of WSNs. While setting a limit on the maximum number of allowable hops is proposed by the paper to address this issue, adding an elementary cluster formation stage and applying the proposed scheme into cluster boundaries is another solution, which also improves the scalability of the algorithm.

In [33], Cluster-based Energy-efficient Scheme (CES) is presented for electing a cluster-head in 2-hop neighbourhood region. The paper introduces a parameter called 2-density of a node. The 2-density of a node represents the ratio between the number of links in its 2-hop neighbourhood (links between the node and its neighbours and links between two 2-hop neighbours of the node) and the 2-hop degree of the node. In CES, each sensor calculates its weight based on 2-density, its residual energy, and its mobility and broadcasts it to its 2-hop neighbourhood and the sensor node having the greatest weight in its 2-hop neighbourhood is chosen as the cluster-head for the current round. The scheme assumes that sensors have 2-hop knowledge and operate asynchronously without a centralized controller. The CLUBS [34] is another algorithm that forms clusters through local broadcast and converge in a time proportional to the local density of the nodes. To select CHs enough far from each other, the algorithm restart the CH selection process for the clusters with CHs within 1-hop range of each other. While local selection of CHs through broadcasting messages is easy to implement and scalable, it delays the convergence of clustering process and may not guarantee well distribution of the CHs throughout the network.

### 2.2. Assisted Schemes

There are numerous advantages to using distributed cluster-based algorithms, but since a single node does not have a general understanding of the topology and characteristics of the entire network, distributed schemes provide no guarantee either about the fair placement of CHs or about the number of CHs selected within the network. Moreover, heuristic algorithms impose transmission of large number of control messages on restricted resources of sensor nodes, which decreases the overall network lifetime. In this respect, *Bs assisted* and *CHs assisted schemes* are proposed to provide fair placement of the optimum number of CHs and to mitigate energy expenditure of nodes in re-clustering stages of a balanced cluster.

#### 2.2.1. BS Assisted Schemes

The inexhaustible resources of energy and high processing capabilities of BS are considered as a powerful and reliable source for sensor nodes to which they can shift the burden of CH selection and cluster formation phases. This also improves the capabilities of end-user to control the placement and number of CHs through the BS in accordance with the characteristics of the network and type of applications. However, these entail the periodic update of the BS with necessary information by sensor nodes.

##### 2.2.1.1. Fair Placement of CHs

To address the shortcomings of LEACH concerning the placement and number of CH nodes, a centralized version of LEACH called LEACH-Centralized (LEACH-C) was presented by Heinzelman *et al.* in [23]. In the setup-phase of LEACH-C, each node transmits its location and energy level to the BS, and the average energy level of the network is calculated by the BS and nodes having a remaining energy level below this average cannot be CHs for that round. The initial stages in [41] are also is the same as LEACH-C, but after the initial cluster formation phase, the clusters are steady and the CH role rotates among the nodes with remaining energy over the average energy of the nodes within the cluster. The centralized selection of CHs ensures that energy load is evenly distributed among all the nodes by selecting a predefined number of CHs and dividing the network into optimum equal size clusters.

Due to the fact that the sensor nodes spend a great proportion of their overall energy in communications, the K-Means Like Minimum Mean Distance Algorithm (KMMDA) proposed in [42] improves the network lifetime by using the K-means algorithm [43] to calculate the minimum mean distance of the nodes as a parameter in CH selection phase. Like other centralized algorithms, the first step in selection of CHs is to transmit the necessary characteristic information of nodes to the BS, while this information, in KMMDA, is the position of the nodes, which is determined by GPS receivers. In the next step, the BS with its high processing capabilities and unlimited source of energy calculates the distances between all the sensor nodes, and the mean distance to all other sensor nodes for each sensor node; and thus the nodes with minimum mean distance are selected for the CH role. The same algorithm is also proposed in [44] that the BS, in addition to finding the mean distance of the nodes, determines zone areas with the diameter less than a threshold value in which two or more nodes are located. Due to sensing area overlap, only one node in active state in each typical zone is sufficient to sense the environment and other nodes within the zone are scheduled by the BS to sleep in the current round. The sensing node duty rotates among other nodes for the subsequent rounds.

The paper [45] also selects CHs according to the mean distance of the nodes from each other. However, the algorithm first divides the network area into several equal parts based on their location information and distances of the nodes, and then the BS calculates the probability of the nodes for CH role according to the ratio of remained energy and the mean distance of the node from other cluster members. Instead of using localizing devices, the paper proposes to use two nodes with determined position and sink as reference points to locate the sensor nodes within the field. After formation of the network, each node broadcasts a signal and two selected nodes calculate the distances of the nodes with regard of the received signal strength and transmit the gathered information to the BS. By updating the table of distances generated by the BS with the received information from two selected nodes, the BS can calculate the distance of every node from others.

The paper [46] concentrates on the selection of CHs based on the traffic density of nodes. In this scheme, BS receives Hello packets from nodes and computes the traffic of each node using a trajectory-clustering algorithm. The nodes participating in the data relaying process towards the BS are considered as CHs. Then the BS splits the network into equal size clusters and broadcasts a message including the selected CHs and their cluster members to all nodes.

##### 2.2.1.2. Optimum Number of CHs

The number of CHs is a determinant factor in the performance of cluster-based algorithms. There are two issues related to this subject, first the optimum number of CHs (K_opt_) in a network consisting of *N* nodes, and second the variation in number of selected CHs per round around the expected value.

Regarding the first issue, the optimal value of *k* is analytically determined in LEACH using the computation and communication energy models. By setting the derivative of total dissipated energies by CH in receiving signals, aggregating data packets and transmission of the aggregated data to the BS with respect to *k* to zero, the optimum number of CHs in an M×M area consisting of *N* nodes is achieved in Equation (11):

(11) $$K_{\text{opt}}=\frac{\sqrt{N}}{\sqrt{2\pi }}\sqrt{\frac{{ɛ}_{fs}}{{ɛ}_{mp}}}\frac{M}{d_{BS}^{2}}$$

According to the analytical Equation (11) and simulation results reported in [23], the optimal number of CHs for a 100-node network is proposed to be around 3–5. In other words, the desired percentage of CHs in LEACH threshold equation to achieve the optimum network lifetime and performance is around 5 percent. However, it should be noted that the percentage proposed by [23] is calculated based on the direct communication of CHs with the BS and therefore, for the schemes that use multi-hop transmission to transfer the aggregated data of CHs to the BS or the schemes with different energy consumption patterns may vary.

In LEACH, number of selected CHs in each round is not constant and it varies over a great range. Although the threshold value T(n) in LEACH is proposed to adjust the desired number of CHs per round, its inherent probabilistic nature causes variations in the number of selected CHs per round, and even some rounds may exist where none of the nodes are selected as CH. The consequences of these variations are a significant loss of network stability and performance.

Although the number of CHs can be easily controlled by the BS in centralized schemes, few researchers address this issue in distributed schemes by supervising the number of selected CHs with assistance of the BS. The authors in [47] address the issue of selection of no CH in a specific round by calling the round invalid and moving the system to the next round without going through the cluster organizing and message transmission phases. The authors of [48] propose splitting the CH selection phase into an initial selection phase and an add-on selection phase. In the proposed scheme, if the number of selected CHs in the initial stage is below some predefined threshold, an add-on stage is called until number of selected CHs exceeds the threshold value. In [49], a semi-centralized scheme is proposed in which the selection of CHs is distributed and self-organized, while the BS controls the number of selected CHs and stops the CH selection phase as the number of selected CHs reaches the predefined optimal value.

While the number of nodes is usually considered constant, there are cases where new nodes should be added to the network or conversely, some of nodes die with the increase in the number of rounds. In these cases, the number of CHs should be dynamic to ensure network energy efficiency, network robustness and the adaptability of the system to the quality of the network. The variation in the optimum desired numbers of CHs per round due to changes in the number of nodes within the network is highlighted by [50]. This paper proposes that CHs send the number of their cluster members with aggregated data to the BS, whereby the optimum number of CHs in accordance with the updated total number of nodes within the field is calculated for the next round.

#### 2.2.2. CHs Assisted Schemes

CHs can collect the up-to-date states of their cluster members through continual communications in data transmission phases. Using this information, CHs can assist in the selection of the next round CHs to balance the clusters and to eliminate extra energy expenditures in re-clustering stages.

##### 2.2.2.1. Balanced Cluster

**Figure 4.**
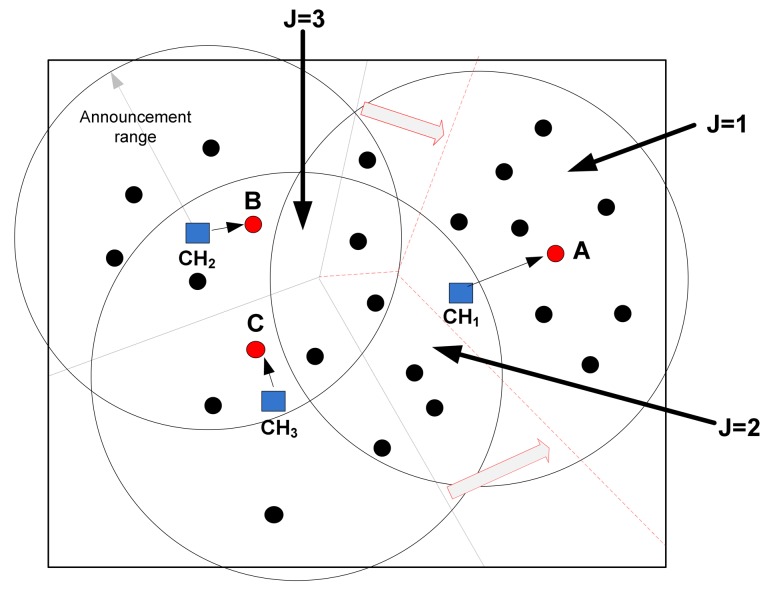
Selection of the next CHs towards balancing the cluster size.

Kim, *et al.* in [51] proposed a new method of CH selection according to the number of nodes in the cluster and the number of CHs within the transmission range of the nodes. The main goal of the presented algorithm is to fairly distribute the CHs and balance the cluster sizes. Like other cluster based algorithms, selected CHs broadcast advertisement messages to announce their selection during the setup phase and then each cluster area is divided into several *sub-regions* using the number of received advertisement messages (*J*). In other words, *J* is the number of CHs within the transmission range of a node and the algorithm advocates the idea that the nodes with the same value of *J* are located in the same sub-regions. During the steady-state phase, each node transmits the sensed data including *J* and the index of its remaining energy to its CH. The current CH selects the next-round CH towards balancing the cluster size by comparing the number of cluster members with the average size of the cluster. If the cluster size is larger than the average size of clusters, the current CH selects the subsequent CH amongst the nodes having the most remaining energy and with *J* value equals to one. In this way, the number of cluster members in large clusters reduces for the next round. From the perspective of the nodes with *J* larger than one, which means the nodes located in the sub-regions closer to the boundaries of the cluster, and the next round's CH moves away from them. Thus, they have greater chance for the subsequent round to join neighbouring clusters. This leads to a decrease in the cluster size. On the other hand, if the cluster size is smaller than the average size of clusters, the current CH selects the next CH from the same sub-region where it is located. This is because of the fact that there are neighbouring CHs having more number of cluster members and according to the previous case, the next CHs in the neighbouring clusters will be selected in a way that the nodes in the non-exclusive sub-regions of the larger cluster will join the smaller cluster for the next round. In the example shown in Figure 4, CH_1_ is located in the area of *J* = 2 and its cluster size is larger than other two clusters. Therefore, for the next round CH_1_ selects node *A* from area of *J* = 1 to move the cluster boundaries towards making the cluster size smaller, while CH_2_ and CH_3_ select nodes B and C from the same *J* areas, respectively. In this way, without imposing extra traffic or complex computations on the network, the algorithm balances the size of clusters and their traffic loads, and thus increases the network lifetime.

##### 2.2.2.2. Energy Expenditure Mitigation in Re-Clustering

Although re-clustering is proposed to increase network lifetime by distributing the heavy load of CH roles among the sensor nodes evenly, re-clustering itself is an energy consuming procedure, which imposes extra transmission of control messages on the network. Re-clustering also hinders the real-time transmission of data at initial stages of each round. In this respect, some solutions are proposed in papers [52–62] to mitigate the issues pertaining to re-clustering while exploiting the positive aspects of round-based clustering schemes.

In [52], a selective CH selection scheme is proposed to minimize the energy dissipation by reducing the frequent communication of conserved nodes in previous cluster with assistance of the current CH. The paper highlights the idea that a great proportions of the nodes around the previous CH have a high possibility of belonging to the same cluster formed by the new CH, therefore, the unchanged nodes of the former cluster can be eliminated from the setup phase and set to sleep mode instead, while only newly added nodes exchange necessary control packets. To select effective CH, besides two common factors of distance and residual energy, the paper proposes to use two other factors of density and transmission cost (*cost*). Both factors are calculated by the number of neighbouring nodes (*Node _neighbour_*) in the same cluster and the number of foreign nodes (*Node _foreign_*) located in other clusters, as presented in Equations (12) and (13):

(12) $$\text{Density}=\frac{{\text{Node}}_{\text{neighbour}}}{{\text{Node}}_{\text{neighbour}}+{\text{Node}}_{\text{foreign}}}$$

(13) $$\text{Cost}=\frac{1}{\alpha_{c}.\text{Density}+\left(1-\alpha_{c}\right).\frac{E_{r}}{E_{\max}}}$$

After selection of new CH based on the aforementioned factors, the current CH provides the information of the selected CH to the cluster members and each node determines its status using this information to learn whether its cluster is changed or not.

The papers [53–56] address the issue by eliminating dispensable re-clustering rounds by replacing the time-based CH role rotation in LEACH algorithm with an energy-threshold-triggering scheme. LEACH-ET [53] triggers the re-clustering phase on a network scale whenever any of CHs' energy levels drops below the predefined threshold level. The algorithm presumes that each node calculates and transmits *n* bits with *P* probability in every round and thus the ET is calculated as *n.p.E_CH_*, where E_CH_ is the energy dissipation rate of the CH per bit. The BS calculates and broadcasts the ET in initial round and each node records the value, accordingly. Hong *et al.* in [54] also propose using of energy threshold for triggering the re-clustering phase, but only CHs having remaining energy level below the threshold value participate in the CH selection process and other CHs ignore this stage and continue as CH for the subsequent round. Therefore, a great proportion of energy expenditure of CHs having energy level above the threshold level is saved.

Instead of demolishing all the constructed clusters, the authors of [55] propose that after the initial cluster construction phase, the cluster boundaries remain constant for a predefined number of rounds, but instead, the responsibility of CH role keeps rotating randomly among the nodes within the cluster until the next scheduled re-clustering round. In RRCH [56] and LEACH-F [23] similar solutions are used that distribute the CH role among the nodes of each cluster. Instead of random rotation of the CH role, the rotation sequence in RRCH and LEACH-F is coordinated either by the CHs or by the BS, respectively. In this way, the energy consumption is balanced among the sensor nodes and the coordinator can select the next round CH based on the energy metrics or any other criteria desired by the user. The only difference of RRCH and LEACH-F concerns the coordinator node that is responsible for sending the sequence numbers to the cluster members, which is CH in RRCH and BS in LEACH-F.

Another approach is Energy Balanced Clustering (EBC), in which re-clustering decisions are correlated to the traffic load processed by the CH in a round. According to the paper [57], re-clustering happens based on the burden of traffic load on CH during each round, not based on a predefined time schedule. The paper advocates the idea of restricting the number of re-clustering cycles only to the rounds it is essential and in this way, it saves remarkable proportions of energy resources of nodes spent in re-clustering stages. In this respect, each node sends details of its remaining energy with sensed data to the CH after initial setup phase. When the amount of data received at a CH exceeds a predefined threshold, the current CH selects next round CH based on the energy level of its cluster members and broadcasts a message about the new chosen CH.

The proposed protocols in [58–62] utilize redundant and backup CHs to improve network performance and to decrease extra energy expenditures in re-clustering phases. Selecting two nodes having the highest energy levels as the potential CHs in initial round and assigning one as the premier CH and the other one as the redundant CH is proposed by [58]. According to the algorithm, nodes are ranked regarding their remaining energy and sum of hops from other nodes; the first rank node is selected as the main CH and the node ranked in the next position is assigned as the redundant CH. To determine the degree of sum of hops, both CH candidates use flooding of broadcasting status information. A list of minimum hop distances from all nodes will be generated and transmitted to the BS to calculate the sum of hops of two candidate nodes. The BS selects the optimum nodes as CHs and informs the entire network about their selection. The redundant CH dynamically updates the cluster information with the master CH, to alternate in case of any failure of CH due to damage, attack or energy depletion. The same approach is proposed in [59] with an additional stage that redundant CH periodically sends a beacon message to CH and counts the number of acknowledgments it does not receive from CH. As soon as the fault counter exceeds a given threshold or current CH remaining energy descends a predefined level, the redundant CH takes over the task of master CH.

In [60], the BS selects the Main CHs based on sensor nodes' remaining energy, location and frequency once selected as CH. Then, the main CHs select the Alternative CHs and Vice-CHs. The Main CHs are responsible for inter-cluster data transmission, while Vice-CHs do data collection and aggregation. Upon the decrease of the remaining energy of Main CHs or Vice-CHs to half of the initial energy, Alternate CHs take their place.

### 2.3. Multi-Factor Evaluation Schemes

CH selection phase is the pivotal stage in cluster-based algorithms, which tangibly affects the performance of the network in the data transmission phase. To ensure a reasonable degree of network lifetime and cluster performance, the most desirable nodes in accordance with the type of application, topology of the network and capabilities of sensor nodes should be selected as CHs. In this respect, CH selection should be regarded as a multi-variable-decision issue with complex inter-relation between factors. The proposed solutions for the complicated process of CH selection in multi-factor evaluation systems are Analytical Hierarchy Process (AHP) and Fuzzy Logic Controller (FLC).

#### 2.3.1. Analytical Hierarchy Process

**Figure 5.**
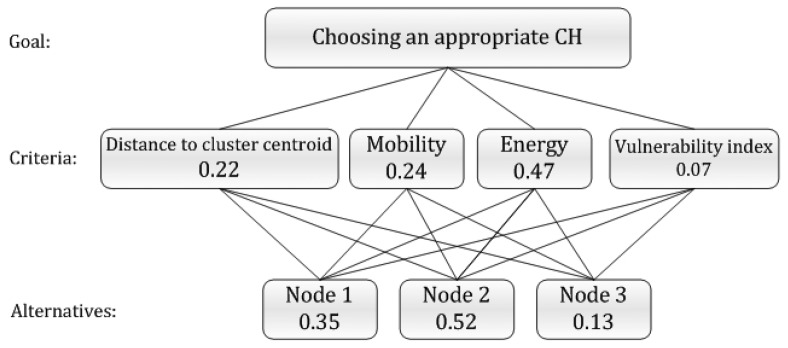
A simple AHP hierarchy consisting of three sample nodes.

AHP [63] is a structured technique for organizing and analyzing complex decisions. In the AHP technique, a complex decision is decomposed into a hierarchy of more easily understood sub-problems using numerical values. At the outset, two matrices should be generated using the fundamental scale for pairwise comparisons: the weight of decision factors towards the topmost goal matrix and the weights of alternative senor nodes towards each factor. A sample table of the evaluated number rating for verbal judgment of preferences is presented in Table 2. Then, the global weight of each sensor node can be obtained through summing the products of the weights of nodes by their corresponding decision factor weights. Thus, the nodes having the largest weight are the most suitable nodes to be selected as CHs. A simple AHP hierarchy consisting of three sample nodes is presented in Figure 5. According to the presented sample, Node 2 alternative's priority with respect to reaching the goal of choosing an appropriate CH is the highest among other nodes; and hence Node 2 is the optimum selection for CH position for current round.

In [64], AHP is used to deal with the issue of CH selection by considering three factors of energy, mobility and distance to the cluster center that contribute to the network lifetime. To minimize the load of data transmission for the nodes which their demises leads to disconnection of the network, the authors of [65] takes into account the vulnerability index of the nodes besides the factors considered in [64]. Therefore, the nodes with higher level of vulnerability have less chance to be selected as CH. For networks with numerous nodes, CH selection using AHP entails solving large dimensions matrices, which should be performed by the BS due to its unlimited energy resources and high processing capabilities.

##### 2.3.1.1. Fuzzy Logic Controller

The authors of [66–76] propose CH selection algorithm based on Fuzzy Logic (FL). FL is a problem-solving control system methodology that provides a simple way to arrive at a definite conclusion based upon a descriptive language to deal with input data more like a human operator. The following compatible features of FL with characteristics of sensor networks make it an apt solution to be implemented in CH selection stage:

- Smooth noise-tolerant output control function in presence of wide range of input variations.
- Adaptive modifiable governing rules for FL controller processes.
- Simple and imprecise implementation of FL keeps the overall system cost and complexity low.
- Reasonable number of inputs can be applied to FL controller.

**Figure 6.**
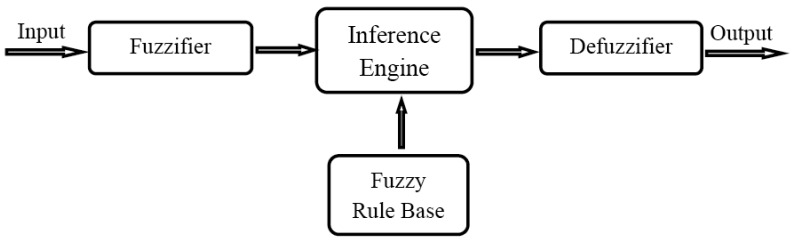
FLC structure.

The basic elements of FLC, shown in Figure 6, are fuzzifier, inference engine, Fuzzy Rule Base (FRB) and defuzzifier. The process of decision-making is performed in four steps:

- Fuzzification of the input variables: taking the crisp inputs from each of them and determining the degree to which the inputs belong to each of the appropriate fuzzy sets.
- Rule evaluation: taking the fuzzified inputs, and applying them to the antecedents of the fuzzy rules. It is then applied to the consequent membership function.
- Aggregation of the rule outputs: the process of unification of the outputs of all rules.
- Defuzzification: the input for the defuzzification process is the aggregate output fuzzy set chance and the output is a single crisp number.

To control FL inputs, fuzzy variables should be defined at the outset. Fuzzy variables are considered as linguistic objects or words rather than the numbers. Each linguistic value is characterized by a label and a semantic value. Triangular and trapezoidal are two common shapes used as input membership functions. Membership function associates a weighting with each of the inputs that are processed; the function also defines functional overlap between inputs and ultimately determines an output response. The rules use the input membership values as weighting factors to determine their influence on the fuzzy output sets of the final output conclusion. Once the functions are inferred, scaled, and combined, they are defuzzified into a crisp output which drives the system. Most of CH selection algorithms using FL adapt Mamdani Method [77] as fuzzy inference technique and Center of Gravity (COG) as defuzzifier approach, but they differ in their utilization of variant input parameters and also in the scope of application of FLC to a local or global perspective of the CH selection process.

In [66], distance of cluster centroid (D), remaining battery power of sensor (SP) and network traffic (NT) are defined as three input linguistic parameters of FLC, while the probability of CH selection (PCHS) is the desired output parameter. The linguistic parameters are defined in Table 3.

The number of linguistic term sets of each parameter is 3; as a result the FRB has 27 rules. According to the paper, the simulations shows higher changes of PCHS by variation of SP input, which means greater importance of remaining battery power of a sensor for the selection of a CH than the two other parameters. In [67,68], the same authors of [66] replace the NT parameter with Degree of Number of Neighbour Nodes (D3N) as another important parameter for the selection of the CH. They evaluate the new proposed sets of parameters and compare the network performances such as network lifetime in [69]. The comparison of two fuzzy-based CH selection systems shows the better performance of the system with D3N as one of the inputs than the previous system. Although considering all the four factors [70] is the optimum solution, it can be concluded that the two parameters of remaining power of sensor nodes and number of neighbour nodes are more important parameters for the selection of CH than distance factor, accordingly.

Gupta [71] introduces FLC for the CH selection phase to reduce the overhead of CH selection. Three fuzzy variables of energy, centrality and concentration of nodes are used to optimize the CH selection procedure and to prolong the network lifetime. As a centralized algorithm, BS collects the necessary information from all the nodes and applies the FLC rules to organize the clusters. The algorithm should address the issues of which other centralized algorithms face too.

To enhance the network expansibility, a distributed Cluster Head Election mechanism using Fuzzy logic (CHEF) is offered by [72] as a contribution to Gupta's algorithm [71]. The initial stage of the scheme is similar to LEACH algorithm, barring the threshold value P_opt_, which is defined as a multiplication of the preferred number of CHs in LEACH by a constant value. According to the value of coefficient, a pool of candidate CHs are selected that each CH uses two fuzzy variables of energy and local distance to elect the optimal CHs. Unlike Gupta's method, CHEF selects CHs in a localized method, which eliminates the overhead of collecting and calculating the fuzzy related information by the BS and ensures selection of one CH within r distance vicinity. However, the calculation cost of FL should be considered as a determinant factor for the exhaustible energy resources of sensor nodes, especially by increasing the number of input variables, which greatly increases fuzzy rules.

Two-level fuzzy decision making is presented in [73], which provides the Local and Global level of decision making. In the local perspective, the algorithm focuses on the physical characteristics of sensor nodes such as internal energy and node degree. However, in the global perspective, network scale factors are considered to achieve balanced and optimized energy consumption. Hence a number of nodes are qualified at the local level by using energy level and number of neighbouring nodes as the input variables applied to local fuzzy system; and then selected candidates are re-evaluated at a global level, based on the parameters of centrality, proximity to the BS and distance between CHs. According to the simulation results presented by the paper, the proposed algorithm outperforms LEACH, Gupta's method and CHEF in terms of network lifetime, residual energy of network and variance of energy in each round, although the complexity and overhead of two-level FL in implementation is not addressed by the paper.

In [76], a Fuzzy Self Clustering Algorithm (FSCA) is proposed as a FL version of ACE [78]. According to the two parts of cluster formation and cluster migration introduced in ACE, FSCA proposes to use two FL modules: Initial Fuzzy Module (IFM) and Migration Fuzzy Module (MFM), which are responsible for initiating new clusters and decreasing the overlap between clusters, respectively. To determine the CHs and to generate cluster sizes equal to or greater than network density, two inputs of node lifetime since the protocol starts and the number of Loyal Followers of the node are applied to IFM in initial stage. Selection of time as one of the inputs is for decreasing the restriction on cluster size to cover the un-clustered nodes in completion of initial stage. By the end of the initial stage, nodes are in one of three states: CH, cluster member or un-clustered. In the migration phase, CHs broadcast a POLL message to their cluster members. By receiving the message, each cluster member counts its Loyal Followers, which are the nodes that are un-clustered or clustered with only one potential CH. By applying the energy level and number of Loyal Followers to MFM, each node calculates its chance to be the next CH and sends it back to the CH, which selects the node having the highest chance for the next round. According to the simulation comparison presented by the paper, the FSCA protocol distributes clusters uniformly throughout the network by migrating close clusters apart, with an advantage over ACE that it also prolongs the network lifetime.

## 3. Cluster Formation

**Figure 7.**
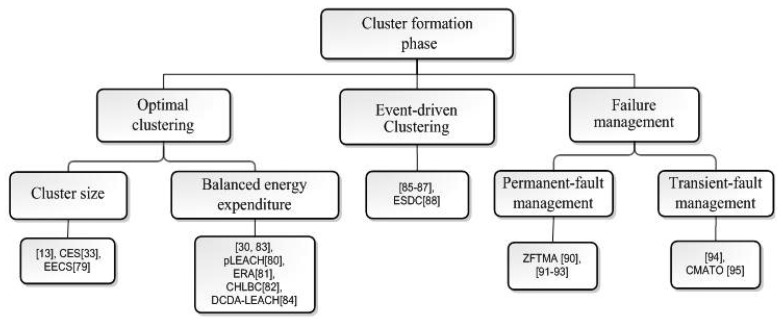
The taxonomy of the cluster formation phase.

Next step after CH selection stage is the cluster formation phase, which starts by broadcasting the advertisement messages by CHs to announce their selection to other nodes, and ends by sending back a join-message to the optimum CH by each node. We group cluster formation schemes into optimal clustering, event-driven clustering and failure management schemes. In optimal clustering schemes, the focus is either to manipulate the size of the clusters according to the type of the application and data transmission, or to minimize and balance the energy expenditure in the network by considering the factors such as data correlation, relay traffic and residual energy. On the other hand, the event-driven clustering schemes are proposed to prolong the network lifetime by eliminating dispensable clustering throughout the network and trigger the cluster formation stage only when and where it is needed, and failure management techniques are discussed to detect faults and recover from failing situations. The taxonomy of cluster formation phase is given in Figure 7.

### 3.1. Optimal Clustering

In the LEACH algorithm, cluster formation is based on minimizing the energy expenditure of cluster members. Sensor nodes join the nearest CHs by calculation of their distance from CHs through the signal strength of the received advertisement messages. This method of clustering does not consider the size of the constructed clusters or even distribution of energy expenditure within the clusters. While most of CH selection schemes accept the cluster formation method proposed by LEACH and they address the clustering issues in CH selection phase, some researchers [13,30,79–84] propose resource-aware algorithms for cluster formation stage separately.

#### 3.1.1. Cluster Size

The papers [13,79] define cluster size as a function of distance to the BS. Based on the type of data transmission from CHs to the BS whether it is direct transmission or multi-hop through other CHs, the number of cluster members in each cluster is manipulated to achieve even energy expenditure within the network. In the Energy Efficient Clustering Scheme (EECS) [79], the original LEACH with 2-hop data transmission is accepted. The energy expenditure of the CHs far from the BS is significantly more in 2-hop transmission, especially in large-scale networks. Therefore, the algorithm justifies the cluster size to balance the load across the network. The cluster sizes of the CHs located farther are smaller than the ones located in close distance to the BS. To do so, the paper proposes a weight function consisting of two factors: node distance to the CH and CH distance from the BS. Using the function each node calculates its cost and joins the CH with the minimum cost. In other words, nodes choose the CH not only based on saving their own energy, but also on balancing the load of the CH which they want to join.

In contrast with 2-hop transmission schemes, the CHs near the BS shoulder the heavy burden of relaying other CHs' data in algorithms using multi-hop transmission for sending data to the sink node. Thus to balance the energy expenditure of CHs, the algorithm in [13] limits the size of the clusters within a minimum and maximum range, based on a linear relation with the distance of the nodes to the BS. Hence, it leads to generating smaller clusters in near distances and larger clusters in far distances to the BS.

To generate balanced clusters, the Cluster-based Energy-efficient Scheme (CES) is proposed by [33], which defines cluster size constraint between the upper and lower threshold. In this scheme, the clusters are constructed by the sensor nodes in 2-hop neighborhood and each node has a generic weight that represents the fitness of the node to be a CH. The thresholds can be chosen arbitrarily, or it can be calculated by the Equations (14) and (15) depending on the network topology:

(14) $${\text{Thresh}}_{\text{Upper}}=\frac{1}{2}\left(\left|N_{12}\left(u\right)\right|+\text{Avg}\right)$$

(15) $${\text{Thresh}}_{\text{Lower}}=\frac{1}{2}\left(\left|N_{12}\left(v\right)\right|+\text{Avg}\right)$$

where *u* and *v* are the nodes with the maximum and minimum of 2-hop neighbors, respectively, *N_12_* is the combined set of one-hop and two-hop neighbors of node and *Avg* is the average number of 2-hop neighbors of all nodes in the network. Based on the threshold values, CH accepts cluster members until the size of the cluster reaches Thresh_Upper_, and afterwards, it drops the messages of the affiliation request. On the other hand, after finishing the setup phase, there may be some clusters which have not attained the cluster size Thresh_Lower_. Therefore, the algorithm calls a re-affiliation phase by the CHs of the clusters whose size is lower than Thresh_Upper_ and higher than Thresh_Lower_. This sort of CHs broadcast the re-affiliation CH message and the nodes belong to small clusters join a new CH based on the received signal strength. The re-clustering procedure in the algorithm is limited only to clusters with lost CHs and the next CH will be selected among other nodes within the cluster. Although the algorithm properly addresses the uneven cluster size without a centralized controller, it cannot consider the optimum number of clusters, because the cluster formation of 2-hop neighbors is prior to CH selection phase.

#### 3.1.2. Balanced Energy Expenditure

The authors of [80] propose a centralized clustering approach to partition the network into an optimal number of sectors and to balance the energy distribution. In partition-based LEACH (pLEACH) [80], the BS is located in the centre of a network field to which each node sends its location and its remaining energy in initialization stage. The BS considers the network as a circular field where every node is marked with the sequence number of its sector according to its central angle. When the amount of data transmission in a sector outstrips other sectors, the BS rotates the partition circle a given angle for the next round to balance the energy dissipation among the sensor nodes.

In the Energy Residue Aware (ERA) clustering algorithm [81], the main goal is to prolong the network lifetime by balancing the energy consumption of the entire network. According to the algorithm, remaining energy and residual energy of each node are two distinct notions. The paper defines the residual energy of a node as its current remaining energy level minus the cost of transmission to the next hop. Therefore, in cluster formation and data transmission phases, each node, either non-CHs or CHs, chooses the next hop not to minimize its power consumption, but rather for forming a route with the maximum sum of the calculated residue energy. In other words, ERA cluster formation scheme emphasizes on even distribution of energy consumption between all nodes rather than the reduction of the average energy consumption of the network.

A dynamic clustering algorithm is presented in Cluster Head Load Balanced Clustering (CHLBC), which considers relay traffic of CHs in clustering stage. In the presented algorithm [82], each CH calculates the relay traffic of a CH either generated by the cluster members or relayed by other CHs in the current round and broadcasts a message containing its ID and level of its relay traffic load across the network area. Therefore, Ordinary nodes decide to join a CH not only based on the distance metric but also difference of the relay traffic load of CHs. The result is that the CHs with a heavier relay traffic load will have smaller numbers of cluster members for the next round than the CHs not actively participating in the procedure of relaying data, and hence the energy consumption of CHs is evenly distributed across the network.

The papers [30,83] define confidence value for CHs according to their characteristics such as remaining energy of the node and CH, distance of CH to the BS and distance of the node to the CH. Each node calculates the confidence value of the CHs in its transmission range and joins the one with the highest confidence value. The confidence value in [30] is calculated by the nodes, using the Equation (16), and is defined in [83] in the form of the summation of three weighted factors, seen in Equation (17). In both Equations (16) and (17), E_r_ is the remaining energy of the node, E_r-CH_ is the remaining energy of CH, D_N-CH_ is distance of the node to CH and D_CH-BS_ is the distance between CH and BS. In Equation (17), MAX_CH-BS_ and MIN_CH-BS_ are the farthest and shortest distance from all the CHs to the BS, respectively and MAX_N-CH_ is the distance of the farthest CH in transmission range of node:

(16) $$\text{Confident}\_\text{valu}e_{CH}=\frac{E_{r}+E_{r-CH}}{{\left({\text{D}}_{N-\text{CH}}+{\text{D}}_{\text{CH}-\text{BS}}\right)}^{2}}$$

(17) $$\text{F}={\text{W}}_{1}\times \frac{E_{i-CH}-E_{r-CH}}{E_{i-CH}}+{\text{W}}_{2}\times \frac{{\text{D}}_{N-\text{CH}}}{{\text{MAX}}_{N-CH}}+{\text{W}}_{3}\times \frac{{\text{D}}_{\text{CH}-\text{BS}}{-\text{MIN}}_{CH-BS}}{{\text{MAX}}_{CH-BS}{-\text{MIN}}_{CH-BS}}$$

As the main objective of clustering is to implement data aggregation within the cluster and thus save energy, the Data Correlation and Data Aggregation LEACH (DCDA-LEACH) [84] algorithm considers data correlation in the cluster formation phase. To find the data correlation of the nodes, the BS explores the data relevance of each node in the first round and divides the network area into a number of data-related areas. Therefore, clustering happens within each area, through dividing the regions into several belt sectors based on the distance of nodes to the BS and the predefined angle threshold. The algorithm forms fan-shaped clusters with high proportions of data correlation.

### 3.2. Event-Driven Clustering

Numerous researchers have focused on generating energy-efficient clusters; most of these solutions offer pre-event clustering and pro-active routing algorithms. However, cluster formation in the entire field prior to occurrence of an event imposes a significant overhead in terms of energy and processing on the network, while it does not guarantee the better performance of the network in some applications. Hence, event-driven clustering solutions are proposed by [85–88]. While the cluster formation procedure and performance in [85–87] is not evaluated in details, a comprehensive event-based clustering algorithm, called Event-to-Sink Directed Clustering (ESDC), is presented by Bereketli and Akan in [88], which considers both event location and direction of data flow from event area towards the sink node. In ESDC, clustering is triggered only within the event region and right after the detection of an event. The clustering continues in a corridor along the data-forwarding path, from the event region towards the BS. Another advantage of the algorithm is in its data transmission towards the sink node. Since the CHs of the event region and the ones located in the path towards the BS are responsible for relaying the generated data, the upstream node in each cluster is selected as the optimum CH of the cluster to minimize the number of data transmissions in data routing. In other words, in ESDC, similar to LEACH-B [89], nodes select a CH having the smallest distance to the BS to prevent data routing back and forth inside the clusters.

### 3.3. Failure Management

The inherent capability constraints of sensor nodes, harsh physical environment and unattended nature of deployment make WSNs vulnerable to failure. In addition, the expectation that WSNs will operate autonomously for a long time necessitates providing fault-tolerant techniques in order to guarantee the network performance and network QoS. The sources of faults in sensor networks may be node-failure due to depletion of battery or destruction by an external event (permanent failure); or it may be link-failure due to environmental condition or medium congestion (transient failure). To overcome the faults, fault-management approaches are applied into networks in three phases: *monitoring, fault detection* and *failure recovery*. In cluster-based schemes, a faulty CH entails isolation of a part of the network; thus, fault-management at CH level is of great importance. In this respect, most of fault-management techniques address the faulty-CH issue. Several investigations propose CH redundancy [58,59,61] (described in Section 2.2.2) as a simple solution for the failure of CHs. However, although CH redundancy is proper mechanism for failure recovery, monitoring and fault detection phases are two other determinant stages that greatly affect the efficiency and latency of dealing with the faults; hence, it is essential to consider fault management as a platform. In addition, the occurrence of fault at other hierarchical levels of cluster is an issue that cannot be addressed only through CH-redundancy. Therefore, several investigations [90–95], which provide fault-management platform for permanent and transient faults in cluster-based schemes, are surveyed in this section.

#### 3.3.1. Permanent-Fault Management

In [90], Zone-based Fault-Tolerant Management Architecture (ZFTMA) is proposed to improve the network efficiency and reliability by adding fault detection and recovery functions as an integral part of the network management systems of the cluster-based algorithms. To minimize resource utilization, the network is divided into four zones; each zone is supervised by Zone Manager node (ZM), which performs as the task manager of the zone in setup phase and is a gateway for forwarding the aggregated data of CHs in steady data transmission state. The CH selection and formation stages of the algorithm are the same as LEACH. However, ZFTMA performs four levels of fault-management, including self-managed CH rotation, sensor node fault-detection, CH node fault detection and CH fault recovery. At the First level, each CH continuously monitors its level of remaining energy. When it drops below a predefined threshold value, the CH selects the highest energized node among its cluster members and announces it as new CH. Sensor node fault-detection level is performed by CHs. The nodes, not sending any packets in a round, are first flagged as suspicious nodes, and after a specific time interval of not hearing from a node, CH announces the node as faulty to the rest of the network. The same procedure is done for CH fault detection level, with a subtle difference that in this level, ZM performs CH fault detection inside its supervised zone and initiates CH fault recovery. At the recovery stage, the algorithm exploits the ZMs' complete view of their zones obtained during data transmission stage by generating lists of all CHs and their cluster members. Having a complete view of the zone topology, ZM selects the new CH among the cluster members of faulty cluster, based on the merit of residual energy. ZFTMA properly addresses node failure at all cluster levels (except ZM) and provides a fault-management platform. However, one drawback of the algorithm is maintaining and transmitting lists of cluster members to ZMs, which imposes extra memory usage and energy expenditure, especially in large-scale networks. Furthermore, the algorithm does not provide any failure recovery strategy for faulty ZMs, thus a faulty ZM may be a bottleneck for the performance of the scheme.

Venkataraman *et al.* [91,92] proposed an energy-efficient distributed cluster-based failure management approach to detect failing nodes and recover the connectivity of network. The main objective of the algorithm is to achieve fast failure recovery with the least overhead, while it only addresses the permanent event failures due to energy exhaustion. The applicability the algorithm is dependent on the special clustering protocol presented by the same author in [96]. According to this clustering scheme, clusters are formed using an expanding ring-search technique. In this technique, CH selects its one-hop neighbouring nodes and each node follows the same procedure for a maximum of *D* number of nodes, until the number of cluster members reaches a predefined maximum number. Based on this technique of clustering and for improving the scalability and manageability of failure detection and recovery, the nodes in clusters are classified into four types: boundary node (has no children), pre-boundary node (whose children are all boundary nodes), internal nodes (has at least one pre-boundary or internal node as a child) and CH node. The failure detection scheme is the same for all four types of nodes through receiving a failure report message generated by a node whose energy level fails below a threshold value, while the threshold value is defined as the energy required for transmission of *D* number of *l-bit* messages across a distance equal to the node's transmission range. However, the failure recovery algorithm is slightly different for each type of node as follows:

1. *Boundary Node Failure Recovery Algorithm*: since the boundary nodes are in fact the leaf nodes, their failure does not affect the connectivity of the network. Therefore, the failure of boundary nodes is simply ignored.
2. *Pre-Boundary Node Failure Recovery Algorithm*: failure of pre-boundary nodes affects the connectivity of its healthy children nodes. Therefore, a healthy child of a failing pre-boundary node follows a procedure for finding a suitable healthy parent by sending a joint message to its neighbors. A healthy boundary child searches for a suitable neighbouring parent having two conditions: neighbor is not among the children of the failing pre-boundary node and the neighbor itself is not a failing node. The healthy child first searches within the cluster and checks whether the supportable degree of the neighbor is within the limit *D*. If no suitable parent found within the cluster, the node searches outside of the cluster considering two limitations numbers *D* and *S* of the new parent and cluster, respectively.
3. *Internal Node Failure Recovery Algorithm*: the healthy child of a failed internal node may be a pre-boundary or another internal node. Both types of healthy children search for suitable parents according to the same procedure in section (2) and if a suitable parent is not found, the child establishes a cluster of its own including all its children. The only difference between parent selection of pre-boundary and internal nodes is in the scope of the search. While the pre-boundary node searches for a new parent either within the cluster or inside the neighbouring clusters, the internal healthy child only searches inside the cluster.
4. *CH Node Failure Recovery Algorithm*: CH failure triggers a new CH selection phase among children of the failing CH based on the merit of energy status. Then, the cluster formation phase is performed within the cluster considering cluster size limits and supportable degree limits.

The analysis presented by the paper shows acceptable level of overhead for the failure detection and recovery mechanism, and the algorithm provides fast failure recovery strategy. However, dependency of the fault-management technique on a specific clustering approach and its single support of permanent faults due to energy depletion limit the applicability of the approach.

Gupta *et al.* [93] proposed a fault-tolerant clustering approach based on an inter-cluster monitoring mechanism. The algorithm addresses the permanent failure of CHs as the bottleneck of cluster-based protocols and provides a fault detection and run-time recovery mechanism using consensus model of the neighbouring CHs to agree on a faulty CH. The fault detection and recovery mechanisms of the approach exploit the time slot feature of the Time Division Multiple-Access (TDMA) MAC protocol by adding two new time slots named “Route Update” and “Status Update”. During *Route Update* slots, cluster members turn on their receivers to receive the new association in the time slot and CH updates; and *Status Update* slot is used by the CH to exchange the latest information about its cluster members and the status of the CH itself with other CHs. The fault detection is done by periodic exchange of *status updates* through inter-CH communication. A CH is considered completely failed only when its *status update* is not received by all other CHs. To answer the CHs' link-failure situation, the algorithm uses the broadcast forwarding of *status updates* by other CHs. However, the forwarding algorithm entails a large amount of redundant message transmission, especially in fault-free fully connected scenarios. The paper addresses this redundancy in two ways. First, a Multiplicative Increase Linear Decrease (MILD) [97] mechanism is applied to the period of the exchange of status updates. The mechanism increases the intervals of *status updates* when there is no fault detected in the network, while it linearly decreases the intervals for faulty networks. Another solution given by the paper to avoid message redundancy in healthy systems is the use of an “*experience*” based model. Using this model, each CH first transmits its experience about its connectivity with other CHs and after receiving the experience of the rest of the CHs, if the network is fully fault-free, no *status update* forwarding happens, otherwise it forwards the updates. Opposite to the comprehensive fault detection technique, the proposed recovery phase by the approach is that the cluster members of the faulty CH simply join the best alternative neighbouring CHs. There are some drawbacks concerning the applicability of the algorithm. First, although the paper applies some techniques to reduce the overhead of the algorithm, periodical exchange of the table of cluster members on a network scale is not an energy-efficient approach for large-scale networks having large-size tables. Another main drawback of the algorithm is that it is primarily designed for scenarios having all CHs in direct communication range with each other. However, this is not the prevailing condition in typical WSNs. Finally, the proposed recovery method is not very efficient and may lead to the creation of isolated regions consisting of orphan cluster members not in the range of any other CHs.

#### 3.3.2. Transient-Fault Management

The authors in [94] propose a smart checkpointing scheme for quick failure detection and recovery of a faulty CH. The paper's contribution is different from its predecessors in term of the type of failure management. While most of fault-management algorithms only address permanent failure [90,93,98,99], the main contribution of [94] concerns detection and recovery of transient faulty-CHs. The scheme selects several additional backup nodes in each cluster during the CH selection phase for checkpointing. The backup nodes are responsible for detecting the status of CHs periodically, and in case of detection of a faulty-CH, one of the backup nodes automatically replaces the CH. To minimize the recovery cost of the network in term of lost-data due to transient CH failure, each CH routinely sends routing information and its collected data to the backup nodes. The level of reliability of the algorithm is dependent on the two factors of checkpointing interval and number of backup nodes in each cluster. To achieve a trade-off between reliability and energy consumption, the algorithm uses a Markov model [100] to determine the minimum number of backup nodes and the optimum value of checkpointing interval, which is the time between two successive checkpoints, while the energy consumption of checkpointing process does not exceed the energy consumption of re-clustering phase due to the CH failure. By solving the Equations (18) and (19), the minimum number of backup nodes (n–1) in a network consisting of *N* nodes and the optimum checkpointing interval (I_ckpt_) can be defined:

(18) $$A_{\text{steady}}=1-\frac{\rho^{n}n!}{\sum_{k=0}^{n}\rho^{k}\frac{n!}{\left(n-k\right)!}}$$

(19) $$I_{\text{ckpt}}\geq \frac{\lambda T}{{\left(N-1\right)}^{2}+2\left(N-1\right)}$$

where A_steady_ (the steady-state availability) is equal to the expected reliability of the user, *ρ* is the ratio of the failure rate (λ) of each node to the repair rate of backup nodes and *n* is the number of backup nodes including the CH. The test-bed implementation and simulation results presented by the paper show that the algorithm saves energy consumption of the network in recovery stage of transient CH failure. Nevertheless, the main achievement of the paper is the significant reduction of recovery latency in comparison to the re-clustering process. While the recovery latency in re-clustering process grows exponentially with the increase of the number of nodes in the cluster, the checkpointing process linearly restricts it with a slight slope.

Cluster-Member-based fAult-Tolerant scheme (CMATO) [95] works at the cluster scale and exploits the overhearing capability of sensor nodes in monitoring the activity of CHs. The objective of the algorithm is to deal with faulty connection of cluster members with their corresponding CH, either due to CH-failure or due to a faulty connection links. In CMATO, cluster members are responsible for detecting faulty CHs by monitoring their links to the CH. There are four states considered for nodes: *error_free, error_detecting, ch_error* and *medium_error*. At the initial stage, all nodes are in *error_free* state by default, until a cluster member finds a faulty-CH through monitoring synchronization beacon messages, packet acknowledgements or MAC layer Wake-up-frames. By detecting a fault, the node enters into an *error_detecting* state and propagates an *unable_list* to the rest of cluster members. The list is propagated and updated among the cluster members, and as the size of the list exceeds a predefined threshold value, the CH is considered as failed and a *ch-fail* message would be broadcast. By receiving the *ch-fail* message, cluster member nodes enter into a *ch_error* state. At this state, the nodes in the range of neighbouring CHs join the nearest CH; the rest of cluster members, not being in communication range of any other CHs, compete for the CH position based on the connectivity factor (the ratio of the number of in-cluster neighbor of the node to the size of cluster) and energy factor. On the other hand, there is another possibility that the generated *unable-list* does not exceed the threshold value. This means that the CH is not faulty, but due to some interference, the communication links of a few nodes are faulty. In this situation, nodes having a faulty medium with a CH join a neighbouring CH or if there is no neighbouring CH in range, they accept an in-cluster neighbouring node having healthy connections with their in-cluster CH as relay hop. CMATO is a distributed algorithm that properly and cooperatively can address the sudden crashes of multiple CHs at running time, and provides fault-tolerance for partial and transient CH failures. However, due to the hostile environment of implementation and inherent unreliability of wireless medium, it is highly likely that temporal disconnection of nodes is interpreted as permanent failure by the algorithm and this leads to frequent CH alternation of cluster members and instability of clusters, accordingly.

## 4. Data Aggregation

In a typical wireless sensor network, a large number of sensor nodes are redundantly scattered to collect the application specific information from the monitoring environment. This redundancy is for two reasons: first, sensing devices are usually low-powered sensor nodes with limited memory, computation ability, sensing and communication ranges. These inherent characteristics of WSNs necessitate a level of redundancy in implementation to achieve the expected level of QoS. Another reason is the common deployment of WSNs in remote and hostile environments, where the possibility of node failure due to environmental conditions is high and each individual node is usually not accessible after deployment. However, this redundancy entails generation of large numbers of highly correlated or even analogous data, which imposes high level of energy expenditure into the network to be processed and forwarded to the BS. Therefore, to save limited battery power of WSNs, Data aggregation mechanisms are proposed.

Data aggregation can be defined as *“a systematic distributed in-network collection, processing and combining of sensed data from several nodes that sense the same phenomenon”*. The main objective of data aggregation is to eliminate redundant transmission and provide fused information to increase network lifetime. Although data aggregation may degrade some QoS characteristics of the network such as latency and data accuracy, achieving a trade-off between energy saving and expected level of QoS can be the proper solution. To achieve the optimal trade-off, data aggregation techniques should be closely coupled with data routing protocols to have complete domination on different forwarding paradigms to promote in-network data aggregation efficiency.

Consequently, data aggregation protocols are usually categorized according to the network architecture as flat and hierarchical (grid, tree, chain and cluster based) techniques. There are couples of existing survey papers on data aggregation [101–104], which investigate several data aggregation techniques including cluster-based protocols. Following the same grouping merit, all the surveyed algorithms in this paper can be considered as cluster-based data aggregation algorithms. since one of the fundamental objectives of cluster-based algorithms is to achieve higher level of energy saving by minimizing transmission costs through data aggregation in CHs and forwarding path. Nevertheless, cluster-based protocols generally focus on improving the routing aspect of protocols and accept simple aggregation operators like MIN, MAX, AVG and XOR to fuse data or to identify the identical received packets [105]. However, in this section, we survey several protocols, which specifically investigate data aggregation process in details in homogeneous cluster-based WSNs. We classify the surveyed cluster-based data aggregation protocols based on the class of data correlation and compression into:

- Spatial: this class exploits the data correlation and redundancy of the readings of a node as a function of the readings at nearby sensors.
- Temporal: in this class, the data correlation of sensor readings as a function of its reading in the past is put to use.
- Spatiotemporal: is the combination of the above two classes. It is interpreted as the utilization of readings correlation of sensor nodes in both spatial and temporal aspects.

**Figure 8.**
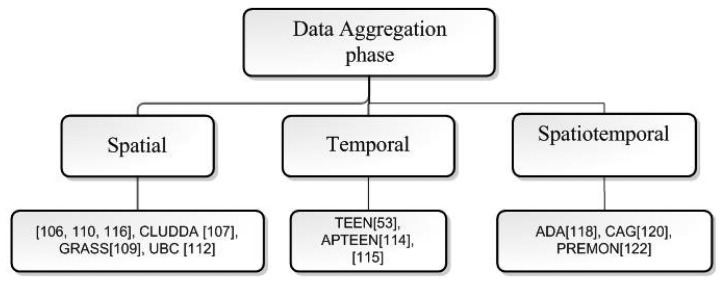
The taxonomy of the data aggregation phase.

The taxonomy of data aggregation mechanisms based on the classes of data correlation is given in Figure 8.

### 4.1. Spatial

Cluster-based data aggregation architecture for Structural Health Monitoring (SHM) is presented by [106]. The paper points out the importance of data aggregation in the SHM application because of the large volume of data generated. The main objective of the paper is to leverage intelligent monitoring by organizing the aggregation method into a three-tier hierarchy. The three-level structure of data aggregation consists of localized computation, data aggregation and distributed computation. In localized computation, every sensor node executes filtering to remove the invalid measurements, and feature extraction to perform pattern recognition of signals and data compression. At the data aggregation stage, the cluster members' data received by CHs go through three steps of filtering, spatial and semantic correlations. While filtering does the same as the previous stage to remove the unreliable results and trivial events, spatial and semantic correlation summarizes the results by their types and explores the contextual relation of different types. The provided regional views of monitored area or objects by parallel clusters are fused at the CH-CH distribution level by location combination, information confidence estimation and inference merging. Filtering is the basic technique used in different levels of the protocol to eliminate noises at the sensor node level and to increase the confidence of the intermediary results at the CH level. Therefore, the paper integrates five filters: threshold filter, deviation filter, quality of information filter, semantic filter and location filter. Through these three steps, raw data generated by each node is condensed into a smaller size and more valuable data until reaching the BS and hence, a great amount of energy is saved in the data communication phase and the reliability of the received information is improved.

The authors of [107] combine Direct Diffusion [108] with clustering and introduce new features of layered data aggregation and dynamic data aggregation points. The so-called Clustered Diffusion with Dynamic Data Aggregation (CLUDDA) protocol constructs the clusters at the initial stage and then uses direct diffusion to route back the aggregated data towards the BS. Although broadcasting of interest messages throughout the network using flooding in Direct Diffusion is an intensive energy consuming operation, the number of nodes involved in this process in clustering protocols is limited to CHs and gateway nodes. To allow the intermediary nodes to do the layered data aggregation, the entire query definitions are provided within interest messages. The queries definitions contain all the required components and describe the operations that need to be performed on components. Each CH or gateway node maintains a query cache including the different data components that were aggregated. This list is used to obtain the final data. Thus using this layered process, data are aggregated in small steps and are reduced along the initial stages of the data propagation path from the node servicing the query towards the node, which propagated the query. Besides, by the change of the location of the source nodes, new CHs or gateways closer to the source which are equipped with the new query definition performs data aggregation and in this way, the aggregation points dynamically change throughout the network, which leads to the even distribution of energy expenditure throughout the network.

Grid-based Routing and Aggregator Selection Protocol (GRASS) is presented in [109] to maximize network lifetime by minimizing the aggregation points while routing data to the BS. The paper combines two issues of the selection of the data aggregation points and the optimal routing route of the aggregated data to the BS, and addresses two issues jointly to achieve the optimum solution. GRASS divides a network into a Virtual Grid Architectures (VGAs). Each VGA consists of several nodes and a CH, which is selected periodically, while the square zones are considered fixed. As a hierarchical approach, CHs performs the first level of data aggregation as Local Aggregators (LA) and a subset of LAs forms the next level of data aggregation, called Master Aggregators (MA). The authors provide two versions of the problem: a two-level scheme and a multi-level scheme. The only difference of two schemes is regarding the data aggregation at MAs level. While in two-level scheme, MAs are not allowed to aggregate the received messages from other MAs, in multi-level scheme MAs are able to do so. GRASS tries to find out the optimal route with selection of the minimum data aggregation points to minimize the maximum power consumption at each LA node by integer linear program formulation and genetic heuristic approach. According to the analytical calculation and simulation results presented by the paper, the network achieves its maximum lifetime extension factor under two-level scheme when the number of MAs reaches the half of the number of selected LAs. In terms of energy-delay tradeoffs a two-level aggregation scheme imposes less reporting delay than a multi-level aggregation scheme, however, it decreases network lifetime contrarily.

To improve the spatial credibility of the aggregated data at CHs, Chang *et al.* [110] introduces fault probability to map dependence weight of each sensor node. According to the protocol, the fault probability of each sensor node is estimated by using a Bayesian Belief Network (BBN) [111], while higher fault probability maps lower dependency weight and *vice versa*. Therefore, a CH by having the fault probability information of its cluster members generates a mapping function of the dependency weight of the cluster nodes, and forwards the aggregated data to the BS only if the accumulated dependency weight of an event collected from sensor nodes in a cluster exceeds a threshold value. This threshold value is calculated by each CH according to the average weight in the cluster, the average number of nodes that sense the event and the average hop counts in a cluster. To answer the special event situations that the accumulated weight of an event is below the threshold value and there is no exactly same event received, the paper also proposes another threshold, called adaptive threshold. Using the adaptive threshold, CH exchanges the average number of sensor nodes that sensed the event into the number of source nodes that the CH received so far. Although the proposed algorithm improves the credibility of the aggregated data, it increases the delay time and memory storage due to the fault information. In this respect, a waiting time is proposed, which starts to count down by receiving of the first event and is formulated via the maximum hop counts in cluster, average computing time, average sensing time and average transmission time.

In Unbalanced clustering (UBC) [112], the significance of the optimal cluster radius according to the aggregation characteristic in a correlated data field is investigated. Given the decrease of the spatial correlation of the generated data of environmental factor in real world along the space field, the main idea of the paper is to find the optimal cluster radius to achieve the most efficient data aggregation ratio. To exploit correlation as an independent coefficient, UBC uses the Discrete Cosine Transform (DCT) [113] as a linear transform, in which statistically spatial-dependent data are mapped into a set of more independent coefficients. Using the statistical results presented by the paper, the trend of the aggregation ratio *versus* cluster radius R is a typical exponential graph. Therefore, the authors approximate the aggregation ratio *Ag_a_* in Equation (20):

(20) $${\text{Ag}}_{a}=\left(1-r\_\min\right)e^{-\pi R^{2}\rho_{d}/\delta_{c}}+r\_\min$$

where *ρ_d_* is the node density in the field, δ_c_ is a constant factor that manages the dropping speed of aggregation ratio and *r_min* is the minimum aggregation convergence point obtained from the statistical results. Using the aggregation model in Equation (20), the optimal cluster radius is different for different regions of the network fields, and this is the reason that the paper proposes to divide the network into unbalanced clusters according to the optimal cluster radius calculated for each region. The simulation results presented by the paper show some improvements in terms of average energy consumption in network *versus* different cluster numbers of equal-size cluster protocols.

### 4.2. Temporal

In some applications such as environment monitoring, only the variation of physical parameters may be required, while in proactive clustering protocols including LEACH-like algorithms, a comprehensive picture of the entire sensing area in each round is provided by the sensor nodes. Therefore, the paper [53] presents a new reactive energy-efficient protocol called Threshold sensitive Energy Efficient sensor Network protocol (TEEN), which defines two Hard Threshold (H_T_) and Soft Threshold (S_v_) values to control triggering of data transmission and to exploit the temporal coherency of sensor readings to suppress data redundancy and reducing energy consumption. In this scheme, each node continuously senses the environment, but it switches on its transmitter to send the sensed data only when the sensed value satisfies two conditions. First, if the current value of the sensed attribute is greater than the hard threshold; and second, if the sensed value differs from the prior record by an amount equal or greater than the soft threshold. Thus, H_T_ reduces number of transmissions by confining the desired value of the sensed attribute; and S_T_ eliminates transmissions of the sensed data when there is minute or no change in the sensed value. By pursuing a hierarchical approach along with the use of data centric mechanism, TEEN is highly responsive to sudden changes in the sensed attributes which is an important feature for time-critical applications. However, the drawback of the method is in situations that the thresholds are not reached and so user may not get any data from network.

To address the aforementioned issue in TEEN and also increase the control level of the user over the network performance, the same authors of the TEEN algorithm proposed Adaptive Periodic Threshold-sensitive Energy Efficient Sensor Network Protocol (APTEEN) [114] as an extension to TEEN. In the proposed algorithm, the best features of proactive and reactive networks are combined and a hybrid protocol is presented which sends data in adjustable time intervals while it still responds to sudden changes in attribute values. To implement the protocol, CHs broadcast a count time (T_C_) in addition to the threshold values to the sensor nodes, which defines the maximum periods between two successive reports by a node; and if a node is not triggered to send data by exceeding the threshold values, it sends the sensed data every T_C_ intervals. Although, due to additional complexity in implementation and further data transmissions, APTEEN's performance in terms of energy dissipation and network lifetime is less than TEEN, it provides additional flexibility and a more complete picture of the network.

A prediction-based data aggregation algorithm is presented in [115] to reduce redundant data transmission. The main objective of the algorithm is to provide a prediction mechanism of data series and to minimize data transmission overhead due to temporal data correlation. To achieve an acceptable level of accuracy, the protocol uses the combination of two data aggregation models: Gray-Model-based Data Aggregation (GMDA) [116] for its merits in quick modeling with a few data items and Kalman-Filter-based Data Aggregation (KFDA) because of its merits in processing data series of noisy measurements [117]. Therefore, Combined Grey model and Kalman Filter Data Aggregation (CoGKDA) can provide high prediction accuracy and more adaptability and scalability to dynamic changes in the distribution of sensed data. The algorithm is based on the synchronization of the prediction data series in the sensor node and the sink node. In other words, at the initial stage according to the level accuracy expected by the user, the sink node broadcasts the acceptable prediction error threshold and cumulative error threshold (accumulation of the error in continuous prediction) to all sensor nodes. Then, the sink node estimates the sensed data of each node at the current period using the received data from previous data sensing period and updates it by receiving new values. At the other end, each sensor node does the same prediction using the same data sequence and saves the predicted values in a queue. Therefore, in the next sensing round, each node compares its sensing value with its predicted value in queue and sends its sensed value only if the error between the predicted value and sensed value exceeds the prediction error threshold. Meanwhile, the sink node by not receiving any sensed value from the sensor node uses its predicted value for the sensing value of this round. However, the continuous of successful predictions faces the protocol with two challenges: distinguishing the difference between not sending data due to successful prediction and node failure, and excessive cumulative error. To address the issues, the protocol exploits another threshold value for the number of continuous and successful predictions, which forces the sensor node to send its sensor readings after exceeding the threshold value. The protocol eliminates redundant data transmission tremendously, while it does not impose any overhead transmission for the algorithm implementation. However, to achieve acceptable level of accuracy, it seems essential to consider a wise trade-off between communication overhead and reducing concurrent error.

### 4.3. Spatiotemporal

Adaptive Data Aggregation (ADA) for clustered WSNs is proposed by [118]. The paper addresses the existing constraints in sensor nodes regarding sensing range, computing and wireless communication capabilities by shifting the main portion of data aggregation to sink node, with a little function at CHs and common nodes. The main objective of the algorithm is to achieve the desired reliability of the aggregated data at the sink node, while limits the imposed cost on the CHs and sensor nodes. In this respect, the paper points out the overlaps of the sensing ranges of sensor nodes in dense networks and addresses this issue at sensor nodes' scale by controlling the reporting frequency (*f*). At CHs level, ADA introduces aggregation ratio (*d*) for spatial redundancy of the sensing data of the same or analogical events received at CHs. Therefore, the paper controls the report frequency and aggregation ratio to improve the observed reliability to the desired level of reliability in the duration of a decision interval (τ). To quantify the level of reliability, ADA defines the total number of received data (*N*) by the sink node as *N = nτf/d*, where n is the number of source nodes. For different applications, according to the desired level of reliability, the sink node sets the f_0_ and d_0_, and the desired and observed reliabilities converge, using a heuristic method.

Yoon and Shahabi in [119,120] provide an algorithm to compute aggregates using CH values. The Cluster AGgregation (CAG) algorithm forms clusters of nodes that sense similar values using the spatial correlation in [119] and the combination of spatial and temporal correlation in [120]. According to the cluster formation stage in CAG, each node decides to join a cluster, based on the level of the correlation of its sensed data, called My local sensor Reading (MR), to the CH sensor Reading (CR) measurement. In other words, considering the user-provided error threshold τ, a node joins a cluster if (|MR − CR| ≤ Range × τ) is satisfied, where *Range* defined as the difference of the *Max* and *Min* value of the entire dataset that is defined by the user or ADC sensor provides. CAG algorithm operation consists of two phases: query and response. During the query phase, the algorithm forms clusters and data forwarding tree, using the same algorithm in Tiny AGgregation (TAG) [121]. Throughout the response phase, CAG transmits a single set of value per cluster. An injected user query with a specified threshold *τ* followed by the broadcast of the query packet initiates the query phase. The broadcasted query packet contains *user query, ParentID, NodeId, Level* (depth of the current node in the forwarding tree) and *CR*. Then in the response phase, the aggregated value is forwarded back along the reverse direction of the query propagation. In situations of two CHs in the path of data forwarding unable to communicate with each other, the algorithm exploits bridge nodes, which do not participate in the aggregation process, and only forward received packets.

Based on the level of data correlation, CAG algorithm is designed for two modes of operation: Interactive mode (spatial correlation) and streaming mode (spatiotemporal correlation). Having two customization modes improve the applicability of the algorithm for variant environments and applications according to the user expectations.

- Interactive mode is well suited for environments with dynamic changes in sensor readings. In this mode, for each user-shot query, the network responds with one set of responses. Thus for scenarios with frequent changes in network's topology or the sensor readings, the spatial correlation of data varies dynamically, and hence new query packets should be initiated each time by the user to repair clusters and the forwarding tree to retain the accuracy of aggregated data within the user-provided threshold.
- Streaming mode, on the other hand, exploits both spatial and temporal correlation of the sensing data to form clusters. This mode is designed especially for less dynamic environments, in which the sensor readings do not fluctuate shortly over wide ranges. While the query phases of both modes are identical, the response phase of streaming mode is its major difference with interactive mode. The query message of streaming mode includes another clause *“epoch duration i”*, which defines the sampling frequency. Therefore, CHs respond to each query message once per epoch, as opposed to the one-time response in interactive mode.

In addition to the number of responses by CHs per each query, there are two other major differences between interactive and streaming modes, which enable streaming mode to produce results with higher accuracy and reliability than interactive mode. First, the aggregated data by each CH is assigned a weight according to the number of nodes within the cluster. Hence, the achieved results using aggregation operators such as AVG is better bounded by the user provided error threshold. Second, to minimize the cluster formation and to keep the accuracy level persistently within the error threshold range, the clusters are dynamically updated and repaired by changes of the sensor readings. In this respect, nodes snoop on the broadcast medium during the response transmission phase; and when the variance of sensor reading value of a node against CR exceeds the allowed clustering range, it leaves the cluster and joins a consistent neighbouring or creates a new cluster with itself. The analytical results of the paper and test-bed implementations of CAG in indoor and outdoor environments show the high level of efficiency and accuracy of the protocol by unifying the query routing and query processing of data aggregation in both spatial and temporal data correlation aspects.

**Figure 9.**
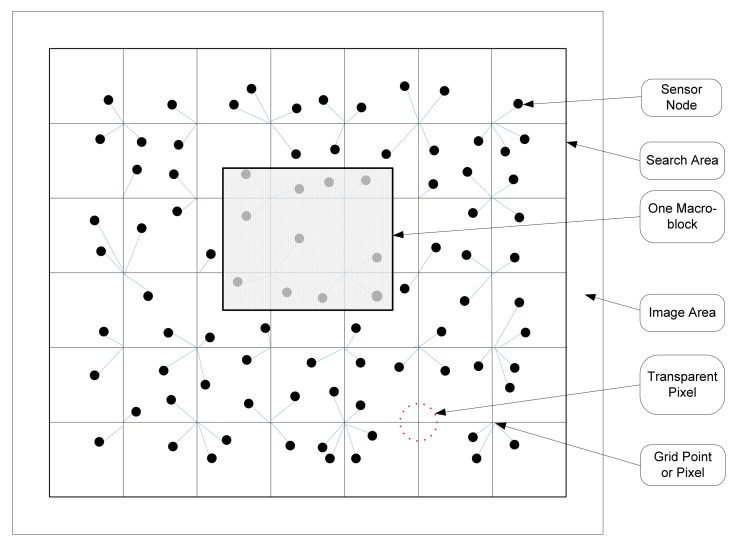
Block matching algorithm: macro block moves to all vertical and horizontal displacements in the search area and data correlation is measured at each block.

With the aim of improving the energy efficiency of monitoring operations of sensor networks, the PREdiction-based MONitoring (PREMON) paradigm is proposed in [122]. PREMON is designed to visualize data aggregation process of WSNs as “*watching of a sensor movie*” and to apply the encoding concepts of MPEG [123] (a standard for audio and video compression) to improve the energy efficiency of network. Based on this analogy, sensor nodes or interpolation of several nodes in a grid point resembles a pixel in an image, while sensor readings are thought as the intensity values of pixels in the image. In addition, one round of monitoring operation corresponds to a snapshot of sensor network as a frame of a movie and the continuous of monitoring process is a continuous of snapshots, which can be interpreted as “*video of sensed values*”. Based on this analogy and considering the fact that sensors in close proximity are likely to have highly correlated readings, the common update-mode sensor-report strategy is improved to a novel prediction based monitoring paradigm. In the PREMON paradigm, based on the spatiotemporal correlation of sensor readings, the BS monitors the readings of sensors for four frames and according to the activity-image of the monitored region, divides the image into macro-blocks (Figure 9) and generates a prediction model using the block-matching algorithm. Meanwhile, nodes follow the update-mode strategy (a technique that at initial round all nodes send a full frame of their readings, and in following rounds, each node transmits its readings only when it is changed) until receiving the prediction model. Then, the BS, using the achieved prediction model, estimates a few frames in future and transmits it to sensors. From now on, each node transmits its reading only when it differs from the predicted value, not when it changes.

There are two differences between the structure of sensor networks and video streaming that affect the performance of data encoding using MPEG encoder in a negative way or positively:

- The most significant difference that degrades the efficiency of algorithm concerns the uneven dispersion of sensor nodes within the network field opposite to the uniform pattern of pixels in a video image. This evenness is a requisite for implementation of block-matching algorithms. To address the issue, PREMON interpolates the readings at regular grid points. In other words, the mechanism assigns readings of sensor nodes to the closest grid point. Grid points with no sensor readings are labeled as “*transparent*” pixels, which match other pixels in encoding process.
- Another major difference is the real-time requirements of sensor networks compared to video encoding. Since real-time expectancy in sensor networks commonly is not as constraint as in video encoding, the prediction model can be generated based on a bigger number of previous images and can be applied for large time-scale patterns. This leads to significant improvements in the accuracy of prediction and thus energy-efficiency. Some other relaxed constraints such as less image resolution and lower frame rate also reduce computation and complexity of implementation of the paradigm in sensor networks.

The results of the test bed implementation of the algorithm, presented in the paper, show the efficiency of the algorithm in cutting down the number of data transmissions and hence achieving great energy savings.

## 5. Data Communication Phase

The main objective of the setup phase is to improve the performance of the network in the data transmission stage. In this phase, CHs as the coordinators of the cluster transmit the aggregated data to the BS for further processing by the end user according to the type of the application. The transmission of a packet from sensor nodes to the CH (intra-cluster) and from CHs to the BS (inter-cluster) can be done through direct transmission (single-hop) or by assistance of other nodes in the path (multi-hop). While several algorithms use single-hop for intra-cluster and inter-cluster data transmission phases, some other schemes claim it inefficient or even infeasible in implementation, especially in scenarios with numerous number of sensor nodes scattered in large area field. However, according to Yin, *et al.* [124], multi-hop transmission is not always the most optimum and energy-efficient solution. By considering the energy transmission model [23] in Equations (21) and (22), the authors of [125] studied the energy-efficiency of single-hop *versus* multi-hop data communication to find the threshold distance of which transition from single-hop to multi-hop transmission leads to saving energy resources. According to the Equation (21), when the distance to the destination meets the d < d_0_ condition, the communication cost between two points is related to the distance by the power of two and it is called short-distance communication. On the other hand, for the condition d ≥ d_0_, the transmission cost is related to the distance by the power of four which is called long-distance transmission. By exceeding the transmission distance from d_0_, the paper [125] calculates in Equation (23) the threshold value d_1_ in which the cost of communication using single-hop outstrips the multi-hop transmission. In this respect, data communication between two nodes can be grouped into three states: single-hop short-distance, single-hop long-distance and multi-hop transmission. While only the cost of transmission of packets are considered in calculation of distance threshold d_1_, to achieve a more accurate threshold value, the cost of transition from sleep mode to active state and processing of packets in relay nodes (RN) are two other factors that should be counted:

(21) $$E_{tx}\left(l,d\right)=\{\begin{array}{c} lE_{\text{elec}}+l{ɛ}_{fs}d^{2},d<d_{0}\\ lE_{\text{elec}}+l{ɛ}_{\text{amp}}d^{4},d\geq d_{0} \end{array}$$

(22) $$E_{rx}\left(k\right)=E_{\text{elec}}\times l$$

where E_elec_ is the energy consumed by the radio transceiver, *l* is the size of the message in bits; *ε_fs_* and *ε_amp_* are the parameters of amplifier energy consumption:

(23) $$d_{0}<d<\sqrt{\frac{\frac{{ɛ}_{fs}}{2{ɛ}_{\text{amp}}}+\sqrt{{\left(\frac{{ɛ}_{fs}}{2{ɛ}_{\text{amp}}}\right)}^{2}+4\times \frac{2E_{\text{elec}}}{{ɛ}_{\text{amp}}}}}{2}}=d_{1}$$

**Figure 10.**
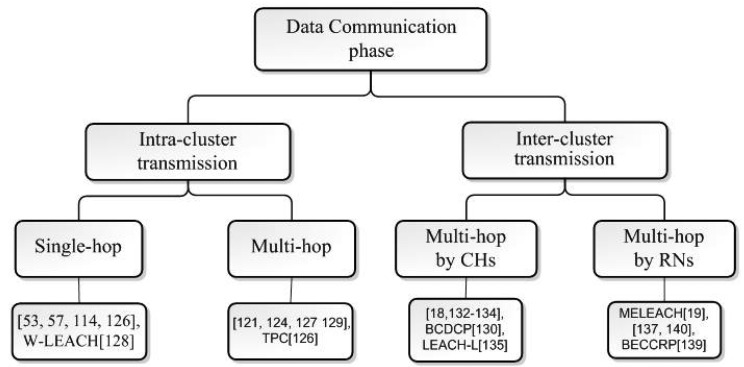
The taxonomy of the data communication phase.

According to the number of hops and type of the nodes used to relay data, we classify the intra-cluster transmission into single-hop and multi-hop transmission schemes and inter-cluster transmission schemes into multi-hop by CHs and multi-hop by RNs. Figure 10 shows the taxonomy of the data communication phase.

### 5.1. Intra-Cluster Transmission

The limited transmission ranges in intra-cluster scale makes both single-hop and multi-hop communication feasible for sensor networks. Some researchers [53,57,114] focus on the advantages of single-hop schemes such as real-time data transmission and less complexity in implementation. On the other hand, some others [124,126,127] count up the disadvantages of using single-hop transmission in large network fields where long distance communication of the nodes located near the boundaries of the cluster may cause radio interference with neighbouring clusters and waste the energy resources of the nodes. However, transmission delay generated in relaying process and construction and maintenance of chains created by the routing protocols in the cluster are two issues that should be addressed by the schemes using multi-hop transmission.

#### 5.1.1. Single-Hop

In the LEACH algorithm, once the clusters are formed, the CH broadcasts a TDMA schedule to cluster member giving the order in which they can transmit their sensed data. One cycle of data transmission by all cluster members is called a frame and every node is dedicated one slot of the frame. After the finish of data communication by the node in the last slot, the schedule is repeated from the first slot until the current round ends. However, the uneven necessity of the nodes to have access to the transmission channel motivates the authors of [57] to address the issue of fixed allocation of TDMA slots to the cluster members by CHs, proposed by the LEACH algorithm. The paper introduces a dynamic TDMA schedule, which adapts to changing load conditions. In the proposed algorithm, sensor nodes are allocated varying time slots according to their traffic in the previous round. Therefore, the nodes with higher rate of data traffic in the current round will be allocated more time slots for the next round, thus they can accommodate high data traffic requirements. On the other hand, the nodes with less data traffic will save their energy with shorter time slots or longer sleeping times. The algorithm saves the energy expenditure of the nodes in idle mode for the nodes with less traffic, while improves the network real-time transmission by the decrease of data-buffering time in sensor nodes with high level of data traffic. However, the paper's contribution is based on the high likelihood of the continuous of the same traffic load of the nodes in current round for the future rounds, which may not be applicable to all scenarios.

Due to the type of implementation, sensor nodes usually scattered non-uniformly within the network field. Weighted LEACH (W-LEACH) [128] is able to handle non-uniform networks and increases the network lifetime. The main contribution of this protocol is to modify the intra-cluster data communication phase of LEACH to make it adaptable to the non-uniform distribution of sensor nodes. W-LEACH assigns weights to each sensor nodes based on the number of its neighbouring sensors (node density of that area) and its remaining energy. The algorithm uses nodes' weights to decide on the competency of the nodes to send data to their CHs. It is important to ensure that sensor readings of the areas having low densities are not overlooked because of fewer numbers of transmitting nodes. To address all the mentioned issues, the weight of each node (w_i_) is defined in Equation (24):

(24) $${\text{w}}_{i}=\left\{ \begin{array}{l} E_{r-i}*d_{i},\;\text{if}d_{i}>d_{\text{thresh}}\\ d_{i}\;\text{otherwise} \end{array}\right.$$

where d_i_ = (1 + number of alive sensors in range r)/(number of alive sensor nodes) is the node density in *r* radius of node *i*, E_r-i_ is remaining energy of node *i*, and d_thresh_ is the density threshold of defining low density areas. Based on Equation (24), the sensor nodes with low weights in each cluster are chosen to send data to the CH. Therefore, the chance of the node from low-density areas to be selected to send their data is more. Using this approach, a uniform data gathering scheme is provided and the level data redundancy decreases extremely. The only drawback of the algorithm is the more frequent selection of the nodes from the areas with node density lower than d_thresh_. This may lead to faster demise of these nodes and hence creation of uncovered areas. The solution presented for this issue by the paper is to sustain data transmission of this type of nodes by some probabilities for some rounds. However, this solution is in contrast with the main objective of the paper and it may result in loss of sensor readings of an area of the network.

#### 5.1.2. Multi-Hop

The protocol presented in [129], also addresses the issue of identical data transmission by the cluster members of a CH. The main objective of the algorithm is to minimize the average energy expenditure of clusters, while it restricts sensing and data transmission tasks to the nodes having higher level of energy in each cluster. To implement the solution, the proposed algorithm uses *Meta-data*, and adds two stages to the data communication phase. Meta-data is defined as an indicator of data type of the source node having smaller packet size than the sensing data. In this respect, the data collection process of the scheme starts by transmission of the meta-data of all cluster members to their corresponding CH. After collection of meta-data, according to the data correlation of the meta-data, the highest energized sensor node among each group of the nodes with identical meta-data is selected by CH as the representative of the group. Then, the CH informs the elected node to transmit the real sensing data and forward the received data to the BS. Performance of the algorithm is dependent on two factors of the ratio of the length between meta-data and sensing data packet, and level of data correlation. However, considering two extra phases of meta-data transmission and representative node selection, the algorithm is only beneficial for the type of applications which monitor phenomena with limited changes and high levels of correlation. A Multi-hop Two-phase clustering (TPC) scheme is proposed in [126] to minimize energy consumption in collecting sensed data while meeting delay constraints. The paper uses multi-hop transmission for sending sensed data to CH to distribute the CH's workload by reducing the number of direct-link communications of a CH with its cluster members. The clustering process is divided into two phases; in the first phase, like other clustering algorithms, the optimum clusters are constructed by CHs and in the second phase, the direct-link star-graph connection between CH and cluster members transforms to a multi-hop data relay communication link. The main idea of the paper is built upon the fact that the sensor nodes are densely deployed within the network field and the generated data of cluster members are highly correlated. Therefore, the data aggregation process can be done in the path towards the CH rather than in CHs only. However, delay constraint is an issue regarding multi-hop transmissions. The paper addresses this issue by using an *n-relay* control message, which is broadcast by CH to cluster members. According to this *n-relay* threshold value and forwarding index of the packet, each node decides whether to transmit the packet to the next RN or directly transmits it to the CH. Moreover, the algorithm implements CDMA/CA (code division multiple access/collision avoidance) as the medium access layer protocol instead of TDMA, because of relatively low cost of implementation and less scheduling problems of the cluster members by CDMA/CA over TDMA in a multi-hop communication algorithm.

Based on the restriction of forwarding angle, an intra-cluster multi-hop routing algorithm is implemented in [124]. In this protocol, an intermediate node is selected according to the following conditions: first, it should satisfy the “triangle inequality”, and second, it should belong to a limited-angle area, which is a restricting angle used to limit the forwarding scope of data packets. In wireless sensor networks, the energy consumption does not always satisfy “triangle inequality”. Therefore, it is important to note that multi-hop transmission is not necessarily an energy saving process. Furthermore, the delay caused by relaying a packet through multiple hops is more than with direct communication. Thus, by considering the energy transmission model in Equation (21), multi-hop transmission is acceptable when the energy condition presented in Equation (25) is satisfied.

Transmission of data from N_1_ to N_3_ through N_2_ reduces the total energy consumption only if the given condition in Equation (25) is satisfied. In other words, the minimal hop path may not be the minimal cost path:

(25) $$\text{E}\left({\text{N}}_{1}{\text{N}}_{3}\right)+\text{E}\left({\text{N}}_{3}{\text{N}}_{2}\right)<\text{E}\left({\text{N}}_{1}{\text{N}}_{2}\right)$$

The selection between multi-hop and single-hop communication of cluster members with CH is done based on the size of the cluster in [127]. The paper defines a critical cluster size (Q_critical_) threshold, which is calculated by CHs according to the network parameters such as number of nodes, size of the network area, number of CHs, transmission energy and energy consumption in data aggregation process (E_DA_). The critical value of the square area size of a single cluster is presented in Equation (26):

(26) $$Q_{\text{critical}}=\frac{E_{TX}+\left(1/P_{CH}-1\right)\cdot \left(E_{RX}+E_{DA}\right)}{{ɛ}_{fs}\cdot \left[P_{CH}-1-1/\pi +\left(1-P_{CH}\right)/\left(2\pi P_{CH}\right)\right]}$$

Each CH calculates the approximate size of the cluster area and if it is smaller than Q_critical_, single-hop communication is implemented; otherwise CH implements the shortest path tree (SPT) within the cluster and cluster members use the routing path to send data packets to the CH.

### 5.2. Inter-Cluster Transmission

The final stage in running a cluster-based algorithm is to route the collected and aggregated data of sensor nodes by their correspondent CHs to the BS, where data are evaluated and processed according to the type of the application. Although single-hop is accepted by LEACH for intra-cluster transmission, many researchers found it infeasible for inter-cluster transmission because of the common long distance communication of CHs with the BS and thus propose implementing multi-hop transmission instead. To implement multi-hop transmission, a backbone should be constructed according to the constraints of WSNs to relay the data towards the BS. The backbone construction should be as simple as possible, energy efficient and applicable, while the total energy consumption of the nodes and the balance distribution of the load among nodes are two other important factors that should be considered. Based on the type of the nodes utilized in creation of the backbone route, we classify multi-hop inter-cluster transmission as multi-hop by CHs and RNs.

#### 5.2.1. Multi-Hop by CHs

A centralized routing protocol called Base-station Controlled Dynamics Clustering Protocol (BCDCP) is proposed by [130] in which the BS carries out all the energy-intensive tasks including CH selection, balanced cluster formation and constructing the routing path. BCDCP is considered as a contribution to the LEACH-C protocol with multi-hop transmission from CH to the BS. BCDCP introduces CH-to-CH routing to transfer data to the base station using minimum spanning tree approach (MST) [131]. The paper addresses two issues in multi-hop data transmission in cluster-based algorithms: the heavy burden of data relaying for the CHs closer to the BS and radio interference caused by the neighbouring clusters. To alleviate the first problem, one CH is randomly selected to forward the data to the BS and thus the burden of routing is evenly distributed among all CHs. The paper also addresses the issue of radio interference caused by neighbouring clusters either in data transmission to CH or in relaying data towards the BS. To counteract the issue, BCDCP utilizes CDMA codes that each CH using its unique spreading code distinguishes the data transmission from the nodes within the boundaries of neighbouring clusters. Thus the CHs without hindering the operation of other clusters, route back the data to the BS. The random selection of a CH to transmit other clusters' data to the BS is modified in [132] that in each round, the CH, which has the maximum remaining energy and minimum distance to the BS, is selected as super CH to relay data.

The weighted spanning tree is introduced in [18] as a backbone to relay data. The algorithm improves the formation of the spanning tree from a single-factor distance-based decision to a multi-criteria one. Selected CHs form a backbone to relay data towards the BS. Therefore, in selection of CHs not only the residual energy and degree of node are considered, but a node's distance to the constructed spanning tree is another determinant factor. The weight of a CH in the spanning tree is evaluated proportional to its remaining energy and the number of its neighbouring nodes, while it is inversely proportional to the distance of the node to the spanning tree route. The algorithm optimizes the inter-cluster data transmission path and extends the network lifetime, especially after demise of half of the nodes within the network. The authors of [133] also propose a weight-based system for selection of relaying path through the CHs. The algorithm calculates the weight of each CH according to the remaining energy of node and its distance to the sink node to construct a routing tree. Although the algorithm may select the optimum next hop from a local perspective, data may be transmitted back and forth in the way of routing to the sink node, which imposes an extra energy expenditure on the network.

An algorithm using multi-layer clustering is presented in [134] that reduces the average distance of each CH from its upper level CH. While cluster formation at the lowest level takes place through a distributed scheme, cluster discovery at the upper levels is done by help of the BS. In each round, the nodes respond to the BS broadcast message at their default low power level with their own ID's and thus, the reply message of the closest CHs only is received by the BS. For the next level, the BS broadcasts another message, which includes the CHID's of the first layer clusters, and nodes reply to this message at their default low power level. Nevertheless, this time the first layer CHs relay received responses to the BS. Following the process until no new CH is discovered, the BS generates a table of the CHID's, level of the CH and ID of the forwarding CHs. In this way, the BS selects the CHs for the lower layer CHs from its immediate upper layer CHs and the algorithm constructs a clustering hierarchy that any node in the given layer reaches the BS in equal number of hops and hence minimizes the energy consumption of the network.

In [135], the authors propose an advance multi-hop routing protocol called LEACH-L as a contribution to LEACH protocol, which substitutes one-hop transmission with multi-hop wise energy-saving selection of relay nodes towards the BS. The paper addresses the issue that although multi-hop routing cuts down the energy consumption in data transmission for every individual node, it increases the circuit energy consumption. Therefore, two thresholds are defined to decide between single-hop and multi-hop transmission from CHs to the BS: the shortest efficient distance for data transmission and Max-distance as the longest distance of direct transmission.

#### 5.2.2. Multi-Hop by RNs

Considering the redundancy of the sensor nodes in WSNs, MELEACH [19] constructs a backbone of sensor nodes which collect data from CHs and transmit the aggregated data to the BS. The paper uses the idea presented in [136] called Energy-aware Virtual Backbone Tree (EVBT) as a scalable algorithm with low overhead to form the backbone. The EVBT algorithm introduces a parameter known as *fitness indicator* to estimate the nodes' competence to join the backbone. The fitness indicator couples the parameters: energy level (e), radio characteristic distance (d_char_) and direction of the link (β ∈ (−π, π)) into a single parameter presented in Equation (27):

(27) $$f_{i}=c_{1}f_{d}+c_{2}f_{e}+c_{3}f_{\beta }$$

where *d_u_* is upstream link length, *f_d_, f_e_* and *f_β_* are fitness parameters defined in Equation (28) and C_1_, C_2_ and C_3_ are the weights of parameters, respectively:

(28) $$\begin{array}{c} f_{d}=1-\min\left(1,\text{abs}\left(d_{u}-d_{\text{char}}\right)/d_{\text{char}}\right)\\ f_{e}=E_{r}\\ f_{\beta }=1-\text{abs}\left(\beta \right)/\pi \end{array}$$

Thus, with the help of EVBT, the MELEACH algorithm constructs the backbone tree route using no-CH nodes, and each CH selects the closest RN as its upstream node to relay its aggregated data to the BS in the data transmission phase.

An adaptive power-aware multi-hop routing algorithm is presented in [137], which defines link costs as a function of node energy and number of hops to find the optimum path to route the CHs' data towards the sink. The cost metric function of the algorithm provides weights for the links based on node residual power, hop count to sink and link quality. Before starting the data communication stage, the sink node lunches a *discovery* message towards the entire network and each node generates a local routing table including its parent node ID and its path cost towards the sink and broadcasts its own *discovery* message. Then, using the graph search Dijkstra's algorithm [138], the nodes update the *path cost* value chosen previously, if it is smaller than the value saved in the local table of the node. The iteration of the paths discovery is dependent on the level of the topology and energy changes of the network, which maintains the costs of the links up-to-date. The simulation results by the paper show improvements in terms of network lifetime and hop-count distance, even though the parameters such as channel delay and interference are not considered. In Balanced Energy Consumption and Cluster-based Routing Protocol (BECCRP) [139], the same factors of number of hops to the sink and energy level are considered to select the gateway nodes and construct the routing path to relay packets. The only difference of this scheme is the precedence in selection of gateways over CHs selection. With the aim of balancing the network load and extending the network lifetime, the nodes firstly compete for gateway position and constructing the multi-hop path to the sink and then the rest of the nodes compete to be as CH.

In [140], the duties of CHs are reduced to path finding and data transmission responsibility is done by gateway nodes. In the cluster formation stage, the network is divided into clusters while the adjacent clusters are overlapped; and the gateway nodes are selected among the nodes located in the overlapped regions. Hence, when a node has data to transfer to the BS, it informs the CH to find the optimum gateway node and CH returns the requested information to the node. However, the paper does not discuss the parameters and conditions of the selection of the gateways and finding the next hop.

## 6. Conclusion and Open Research Issues

Clustering in sensor networks is a hot research area, with a rapidly growing set of research results within recent years. While the ultimate objective behind all the protocols is to prolong the network lifetime and enhance the network performance, each protocol focuses on improving the clustering attributes in a specific phase. In this paper, we have conducted a comprehensive survey of cluster-based routing protocols for homogeneous sensor networks. We focused on the contribution of the routing protocols to each phase of clustering process including CH selection, cluster formation, data aggregation and data communication. We also provided the taxonomy of the protocols in each phase according to their objectives and strategies. Then we reviewed major issues in each phase of clustering process and proposed our suggestions for several issues that deserve more attention. We summarize the taxonomy of cluster-based routing protocols for homogeneous WSNs in Table 4, which provides a summary of objectives, characteristic and issues of every individual scheme and approach of each phase.

The most determinant stage in network performance is the CH selection phase, thus many proposals are presented in the literature suggesting variant CH selection approaches and strategies such as fixed probabilistic, adaptive weight-based, BS or CH assisted and multi-factor evaluation schemes. Handling the heavy tasks like exchanging the control messages, data aggregation and data communication by CHs has led to wide acceptance of CH role rotation strategies. Further research showed that several characteristics of basic clustering schemes such as fixed probabilistic or round robin fashion CH selection schemes, basic cluster formation strategies and direct single-hop data transmission cannot guarantee the optimum performance of the homogeneous sensor networks. Therefore, in depth investigations are done for improving the issues and attributes of basic clustering schemes in the following directions:

- Selectivity: remaining energy level of an individual node, average residual energy of nodes, dissipated energy in last round, number of neighbouring nodes in a defined radius, distance from the BS and sensing coverage are the parameters taken into consideration in the literature to optimize the selection of CHs and thus to improve the network lifetime and performance. However, selection of the nodes having the highest energy level is the most favorable and widely accepted feature among other parameters.
- Even distribution of CHs: guaranteeing the fair dispersion of CHs within the network area is an important issue, which is not provided by the basic clustering schemes. Self-organized schemes address this issue by considering density dispersion parameter in CH selection process or by selecting CHs based on regional merit, although location awareness and learning the resources information of neighbouring nodes are the extra costs in implementation of these schemes. Another solution to ensure the even placement of CHs is the supervision of the CH selection process by generating a global view of the network and controlling the numbers and position of the selected nodes for the CH role by using BS assisted schemes. However in centralized schemes, sensor nodes should be able to reach the BS to provide the necessary information regarding position or their updated resources status. By considering the limited transmission range and battery resources of the sensor nodes, direct communication of all the nodes or even CHs with a BS located in far distances from the sensing area is not feasible in large-scale fields. Furthermore, using multi-hop schemes to transmit this information entails implementation of a temporary routing protocol in initial stages that affects the scalability and real-time operation of the network and increases the algorithm complexity and overhead. Therefore, a trade-off between the costs and benefits of centralized algorithms should be noted.
- Failure recovery: the failure of CH as the coordinator of a cluster causes interruption in performance of a great part of the network. In basic clustering schemes, this failure persists up to next re-clustering round, and this is the reason that the nodes having higher level of energy are selected as CH to minimize the chance of CH failure due to depletion of energy resources in most schemes. However, there are other reasons of failure such as territorial conditions, physical attacks (jamming or tampering) or node mobility. Thus, it seems essential to consider failure-management systems to improve the performance of the network and to reduce the transition time in failure recovery situations.
- Re-clustering: although rotation of CH role is one of the bases of clustering algorithm to distribute the energy consumption among all the nodes, exchange of control messages in clustering process to re-construct the clusters is itself an energy intensive process. Therefore, it is essential to answer the following questions, “when is re-clustering necessary to select the CHs?” and “does re-clustering require re-initiation of complete cluster formation process?” Several proposals are presented in the literature to provide answers for the aforementioned questions such as replacing the time-based clustering process with an energy-threshold-triggering algorithm, elimination of the unchanged nodes of the former cluster from setup phase of next round clustering, rotation of CH role among the cluster members instead of running a new cluster formation process from scratch and event-driven clustering to eliminate the unnecessary clustering overhead in whole regions of network.
- Data aggregation: redundancy in WSNs is necessary to guarantee the level performance of vulnerable sensor nodes. Minimizing this redundancy is one of the main reasons for implementation of cluster-based schemes. Data aggregation in basic clustering algorithms is taken into account only as full aggregation, in which regardless of the number of packets or level of data correlation a single packet is produced by CHs. Nevertheless, this degrades the accuracy and reliability of the aggregated data significantly. Therefore, towards achieving a trade-off between the energy saving and level of accuracy expected by the user, spatial and temporal data correlation should be considered in data aggregation process. Furthermore, it is important to integrate data aggregation into routing protocol to achieve higher data fusion ratio in multi-level data aggregation architecture.
- Application dependency: cluster-based algorithms heavily rely on the type of the application. Therefore, the clustering process is affected by the application needs and specifications to adapt to its variety of requirements. Besides, the inter-relation between the environmental conditions, network topology and the requirements of the application presents the clustering organization as a multi-variable-decision that should be addressed by multi-factor evaluation systems such as AHP and FLC. In other words, these evaluation systems are more flexible and modifiable to the variable range of the application needs and can be easily adapted to the new wants of the user or even changes of goals of the application by subtle modification of the decision rules instead of complete alteration of clustering protocols, which seems infeasible especially in large-scale networks.
- Balanced clustering: cluster formation in basic clustering approaches is based on minimizing the energy expenditure of cluster members by joining the nearest CH. This method of clustering does not guarantee the creation of balanced clusters. Cluster formation can be regarded as a sub-stage of CH selection phase in which constructing balanced clusters is the consequence of the even distribution of CHs. On the other hand, it can be considered as a separate phase, which can manipulate the cluster sizes by implementing resource-aware algorithms and guarantee the balanced expenditure of energy in network field, through defining threshold constraints for cluster sizes or constructing the cluster in K-hop neighbouring region.
- Network connectivity: the clustering scheme has to ensure the intra-cluster and inter-cluster connectivity. The basic clustering schemes assume that cluster members and CHs can directly reach the destination, but given the communication constraints of sensor nodes, the feasibility of establishing the direct route communication especially for inter-cluster data transmission in large-scale scenarios is doubted by researchers. Improving the scheduling algorithm of cluster members in single-hop data transmission and creating a tradeoff between the energy-efficiency and delay caused by multi-hop transmission are the issues addressed by researchers regarding the intra-cluster data transmission schemes. Furthermore, construction and maintenance of a backbone of relaying route through the CHs or RNs are the problems surveyed in the literature to ensure the reliability and efficiency of inter-cluster route.

Although there have been many researches attempting to answer the shortcomings of clustering approaches and improving the characteristics of cluster-based routing protocols, there are several open issues that deserve more attention:

- Most approaches assume the number of CHs calculated in [23] as the optimum value. However, it is important to note that the presented optimum number in the literature is a function of computation and communication energy models for the proposed single-hop clustering protocol. Therefore, when multi-hop data transmission scheme is accepted or clustering protocols with different computational overhead are implemented, the optimum number of CHs should be revised.
- Relay of the aggregated data of clusters is dependent on the performance and availability of the adjacent CHs. Therefore, CH role rotation in neighbouring clusters should be considered as a determinant parameter in CH selection process.
- Topology changes due to the territorial conditions or demise of the sensor nodes is a common situation in implementation of WSNs. Therefore, it is highly needed to study the robustness of clustering protocols against topology changes and to provide reactive solutions to answer inconsistency of clusters.
- Network expandability is another issue that has not been sufficiently surveyed by researchers. In some large-scale applications, it may be favorable to expand the monitoring area with new sensor nodes. The adaptability and scalability of the clustering protocol against the newly added sensor nodes with different resource capability needs to be carefully investigated.
- While permanent-fault management in cluster-based architecture is well studied, transient-fault management due to the temporal link failure needs more investigations.
- Construction of a reliable and efficient relay backbone to route the aggregated data of CHs to the BS using the massively redundant nodes must be further investigated.

## Figures and Tables

**Table 1.** List of symbols in order of the occurrence.

**Symbols**	**Description**	Eq.No.*	**Symbol**	**Description**	Eq.No.*
**T(n)**	Threshold value	(1)	***Avg***	Average number of 2-hopneighbors	(14)
***P****_CH_*	Desired percentage of CHs	(1)	***Thresh****_lower_*	Lower threshold of cluster size	(15)
***r****_c_*	Current round number	(1)	***E****_r-CH_*	Remaining energy of CH	(16)
***E****_c_*	Current energy level	(2)	***D****_N-CH_*	Distance of node to CH	(17)
***E****_i_*	Initial energy level	(2)	***D****_CH-BS_*	Distance of CH to BS	(17)
***r***	Last round number	(4)	***MAX****_N-BS_*	Maximum distance from all CHs to BS	(17)
**E_r-dissipate_**	Node dissipated energy	(4)	***MIN****_N-BS_*	Minimum distance of all CHs to BS	(17)
**E_r_average_**	Node average initial energy	(4)	***MAX****_N-CH_*	The distance of farthest CH in transmission range of node	(17)
**E_r_**	Node remaining energy	(4)	***E****_i-CH_*	Initial energy of CH	(17)
***N****_b_*	Number of neighbors	(5)	***A****_steady_*	Expected level of reliability by user	(18)
***E****_0_*	Initial energy	(5)	***ρ***	Ratio of failure rate to repair rate	(18)
***d****_BS_*	Node distance to BS	(6)	***λ***	Failure rate	(19)
***R***	Cluster radius	(6)	***ρ****_d_*	Node density in the field	(20)
***P****_r_*	Probability	(6)	***δ****_c_*	Constant factor for dropping speed	(20)
***E****_start_*	Initial energy	(7)	***Ag****_a_*	Aggregation ratio	(20)
**α_c_**	Constant weight	(7)	***r_min***	Minimum aggregation convergence point	(20)
***δ***	Time duration of CH selection phase	(7)	***E****_elec_*	Energy of radio transceiver	(21)
***p***	Some random number	(8)	***ε****_fs_*, ***ε****_amp_*	Amplifier energy consumption parameter	(21)
***T***	Predefined maximum time of CH competing duration	(8)	***l***	Size of message in bits	(21)
***j***	Neighboring node ID	(8)	***E****_TX_*	Transmitting energy	(21)
***E****_rj_*	Residual energy of neighbor node j	(8)	***E****_RX_*	Receiving energy	(22)
***d****_BS_*	Distance of nodes to the BS	(9)	***d***	Transmission distance	(23)
***K****_opt_*	Optimum number of CHs	(11)	***w****_i_*	Weight of node	(24)
***N***	Number of nodes	(11)	***Q****_critical_*	Critical cluster size	(26)
***Node****_neighbor_*	Number of neighboring nodes in the same cluster	(12)	***E****_DA_*	Energy consumption of data aggregation process	(27)
***Node****_foreign_*	Number of foreign nodes located in other clusters	(12)	***f****_d_*, ***f****_e_*, ***f****_B_*	Fitness parameters	(27)
***Cost***	Transmission cost	(13)	***d****_char_*	Radio characteristic distance	(28)
***E****_max_*	Maximum energy	(13)	***e***	Energy level	(28)
***Thresh****_upper_*	Upper threshold of cluster size Combined set of 1-hop and 2- hop	(14)	***d****_u_*	Upstream link length	(28)
***N****_12_*	neighbors of node	(14)	**β**	Direction of link between (-π, π)	(28)

* The Equation numbers are according to the first occurrence of symbols.

**Table 2.** A fundamental 1 to 9 scale.

**Number Rating**	**Verbal Judgment of Preferences**
**1**	Equally preferred
**3**	Moderately preferred
**5**	Strongly preferred
**7**	Very strongly preferred
**9**	Extremely preferred
**2, 4, 6, 8 indicate the medium value of above pairwise comparison.**	

**Table 3.** Linguistic parameters and their terms set.

**Linguistic Parameters**	**Term Sets**
**Distance (D)**	Near, Moderate, Far
**Sensor Power (SP)**	Low, Moderate, High
**Network Traffic (NT)**	Light, Moderate, Heavy
**Probability of CH**	Very Weak, Weak, Little Weak, Medium,
**Selection (PCHS)**	Little Strong, Strong, Very Strong

**Table 4.** Summary of the taxonomy of clustering phases.

**Phase**	**Scheme**	**Approach**	**Objectives**	**Characteristics**	**Issues**

**Cluster Head Selection**	Self-organized schemes	Fixed Probability	distribution of energy-intensive CH role among all sensor nods	CH selection based on fixed parameters such as number of CHs, round number, time interval, node ID, location information	Sheer probabilistic, Not considering network resources and parameters

		Adaptive Weigh-based parameters	Distributed selection of the optimum CH based on network parameters or type of application	CH selection based on weighted parameters such as energy expenditure, density dispersion, sensing coverage, regional selectivity, node distances from the BS	Increases setup phase convergence time, Extra overhead, Extra cost of localizing devices or algorithms, Variance in number of selected CHs per round

	Assisted Schemes	BS Assisted	Using inexhaustible resources of energy and high processing capabilities of the BS for optimal selection of CHs	Fair placement of CHs, Limiting the variance of the numbers of selected CHs, Transferring the computing-intensive tasks to the BS side	Periodic update of the BS with the latest information by sensor nodes, Centralized controlling a bottleneck for the network performance and scalability

		CH Assisted	Exploiting the up-to-dated information of CH from its cluster members to assist in selection of next round CH	Balancing the clusters in terms of traffic load and energy, Eliminating extra energy expenditure of constructing new clusters, Restricting the number of re-clustering cycles	Imposes extra overhead to heavy-loaded CHs

	Multi-factor evaluation schemes	AHP	Addressing multi-variable-decision with complex inter-relation of variables in CH selection phase	Decomposing complex decision of CH selection into a hierarchy of more easily understood sub-problem using numerical values	CH selection using AHP entails solving large dimensions matrices for the networks with numerous numbers of nodes

		FLC	Providing a simple way to arrive at a definite conclusion based upon a descriptive language to deal with determinant factors in CH selection	Smooth noise tolerance, Adaptive modifiable rules, Low cost and complexity, More flexible to variable range of applications	List of rules exponentially enlarges by the increase of the number of parameters or number of linguistic term sets of each parameter

**Cluster Formation**	Pre-event clustering	Optimal Clustering	Improving the passive joining the nearest CH method into an optimal cluster construction phase based on network and application conditions	Manipulating the size of clusters to minimize and balance energy expenditure of clusters, Constructing balance clusters throughout the network	Imposes extra overhead in terms of data communication and process on the regions having no event to report

	Reactive clustering	Event-driven Clustering	Limiting the construction of cluster to the regions sensing an event and the path of data flow towards the BS	Cluster formation based on the event location and direction of data flow, Minimizing the energy expenditure in clustering	Not suitable for scenarios that nodes must periodically transmit their sensor readings to the BS

**Cluster Formation**	Failure management	Permanent-fault management	Providing mechanisms for detecting and recovering of permanent node failures	Improving data reliability network performance and QoS Recover isolated part of network due to permanent CH failure	Failure recovery latency, Much focus on CH failure only, Extra transmission overhead

		Transient-fault management	Providing mechanisms for detecting and recovering of transient faulty nodes or link failure	Maintaining level of network performance against temporary failures	Recovery latency for real-time applications, Distinguishing between permanent and transient failure

**Data Aggregation**	Cluster-based and hierarchical	Spatial	Decreasing data redundancy using spatial data correlation, Increasing the data aggregation ratio	Filtering redundant data, Layered data aggregation, Optimum selection of aggregation point to improve aggregation efficiency, Optimal cluster radius to achieve the most efficient data aggregation ratio	Degrades credibility of the aggregated data, Increases delay time and memory storage

		Temporal	Reducing data transmission by exploiting the data correlation of sensor readings as a function of its reading in the past	Extending nodes' lifetime through minimizing the frequency of sensor readings transmission and using temporal-prediction model to estimate sensed data of nodes in future rounds	Reduces the responsiveness of the network to sudden changes of sensor reading values, Degrades fault detection mechanism of network

		Spatio-temporal	Achieving higher aggregation ratio by the utilization of readings correlation of sensor nodes in both spatial and temporal aspects	Exploiting the benefits of the both spatial and temporal data correlation, Achieving high energy saving	The issues of both spatial and temporal schemes

**Data Communication**	Intra-cluster transmission	Single-hop	Providing direct reliable medium with the least interference for transmission of meta-data to the CH	Real-time data transmission, Less complexity in implementation, Easy nodes' scheduling, Less buffering time	Depletes nodes' battery power for long-distance data transmission in large-scale networks

		Multi-hop	Mitigating nodes' energy expenditure in direct long-distance data transmission	Reducing direct link communication, on path data aggregation, finding minimal hop path	Increases the delay of data transmission phase, might not be always energy conservative

**Data Communication**	Inter-cluster transmission	Multi-hop by CHs	Using neighboring CHs to relay the aggregated data of the CH towards the BS	Altering Long distance communication of CHs and BS, Constructing a backbone of CHs to relay CHs' data, Using CDMA to alleviate radio interference	Adds extra overhead to heavy-loaded CHs, Depletes the CHs closer to the BS faster, CH-CH communication might be long distance transmission for large-scale networks

		Multi-hop by RNs	Constructing a relay backbone consisting of the redundant nodes to relay the aggregated data of CHs and performs in-network data aggregation	Restricting the CHs communications to a short distance data transmission with relay node, Selecting optimum relay nodes	Construction of relay backbone imposes extra overhead to the network,
